# Beyond PEG: Emerging Polymer Lipid Alternatives for Lipid Nanoparticle (LNP) Formulations

**DOI:** 10.1002/advs.77145

**Published:** 2026-08-12

**Authors:** Zihnil A. I. Mazrad, Yi Ju, Stephen J. Kent, Colin W. Pouton, Kristian Kempe

**Affiliations:** ^1^ Drug Delivery Disposition and Dynamics Monash Institute of Pharmaceutical Sciences Monash University Parkville Victoria Australia; ^2^ Materials Science and Engineering Monash University Clayton Victoria Australia; ^3^ Olivia Newton‐John Cancer Research Institute and School of Cancer Medicine La Trobe University Heidelberg Victoria Australia; ^4^ Department of Microbiology and Immunology Peter Doherty Institute for Infection and Immunity The University of Melbourne Melbourne Victoria Australia; ^5^ Melbourne Sexual Health Centre and Department of Infectious Diseases Alfred Hospital and Central Clinical School Monash University Melbourne Victoria Australia

**Keywords:** lipid nanoparticles, mRNA, nanomedicines, Poly(ethylene glycol) alternatives, stealth polymers

## Abstract

Lipid nanoparticles (LNPs) have rapidly emerged as the leading delivery platform for nucleic acid therapeutics due to their high encapsulation efficiency, scalable manufacturing, and clinical success in siRNA and mRNA medicines. Poly(ethylene glycol)‐lipids ((PEG)–lipids) have been central to this progress by providing steric stabilization, size control, and tunable biodistribution. However, PEGylation also introduces important limitations, including complement activation, pre‐existing and treatment‐induced anti‐PEG antibodies, and accelerated blood clearance, which increasingly constrain repeated and long‐term dosing strategies. This review focuses on recent advances in PEG‐alternative LNP designs, including non‐PEG polymers, zwitterionic and biomimetic lipids, polypeptides, and structurally modified PEG analogues. We compare how polymer chemistry, anchor geometry, and grafting architecture influence LNP formation, physicochemical properties, biodistribution, cellular uptake, and immunological outcomes. Moreover, we discuss key challenges that remain in translating PEG‐free and PEG‐modified LNPs toward clinical application and propose future directions to better understand in vivo behavior and enable rational design of next‐generation stealth LNPs. Overall, PEG lipid alternatives should not be viewed as simple PEG mimics, but as distinct surface‐engineering materials that create new nano‐bio interfaces and reshape LNP behavior in biological systems.

## Graphical abstract

This review discusses recent advances in PEG‐alternative LNP designs, including non‐PEG polymers, zwitterionic lipids, and polypeptides. It evaluates how surface‐engineering chemistry influences LNP formation and biological behavior, and highlights current limitations and opportunities of PEG‐alternative LNPs.

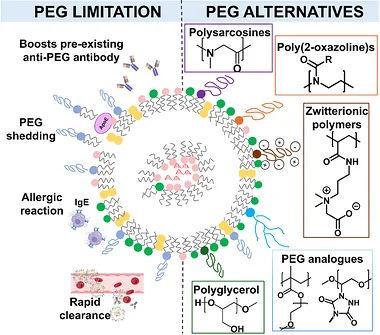

## 1 Introduction

LNPs have become leading carriers for nucleic acid therapeutics, due to their high encapsulation efficiency, biocompatibility, and clinical scalability [1]. Structurally, LNPs are composed of ionizable lipids, helper lipids, cholesterol, and hydrophilic polymer conjugated lipids, each fulfilling a specific task, but overall contributing to particle stability, biocompatibility, and efficient intracellular delivery. The continuous success of these LNPs has demonstrated their enormous potential as next‐generation drug delivery systems, evidenced by the exponential increase of publications since 1990s. Publication numbers increased dramatically from 23 publications in 1996 to more than 3000 in 2020 [2]. The transformative clinical success of LNPs has been exemplified by mRNA‐based COVID‐19 vaccines and by ONPATTRO (patisiran), the first approved LNP‐formulated siRNA therapy [3].

In these systems, poly(ethylene glycol) (PEG)–lipids (PEG‐lipids) play a critical role in controlling particle size and biodistribution, which is influenced by the stability of PEG–lipid anchoring at the surface of LNPs. Despite their central role in clinically successful LNP formulations, PEG itself is increasingly associated with limitations such as immunogenicity, accelerated blood clearance (ABC) upon repeated dosing, and reduced intracellular delivery efficiency [4, 5]. These challenges have prompted growing interest in the development of alternative stealth strategies to replace or supplement PEG in LNP surface design.

**FIGURE 1:**
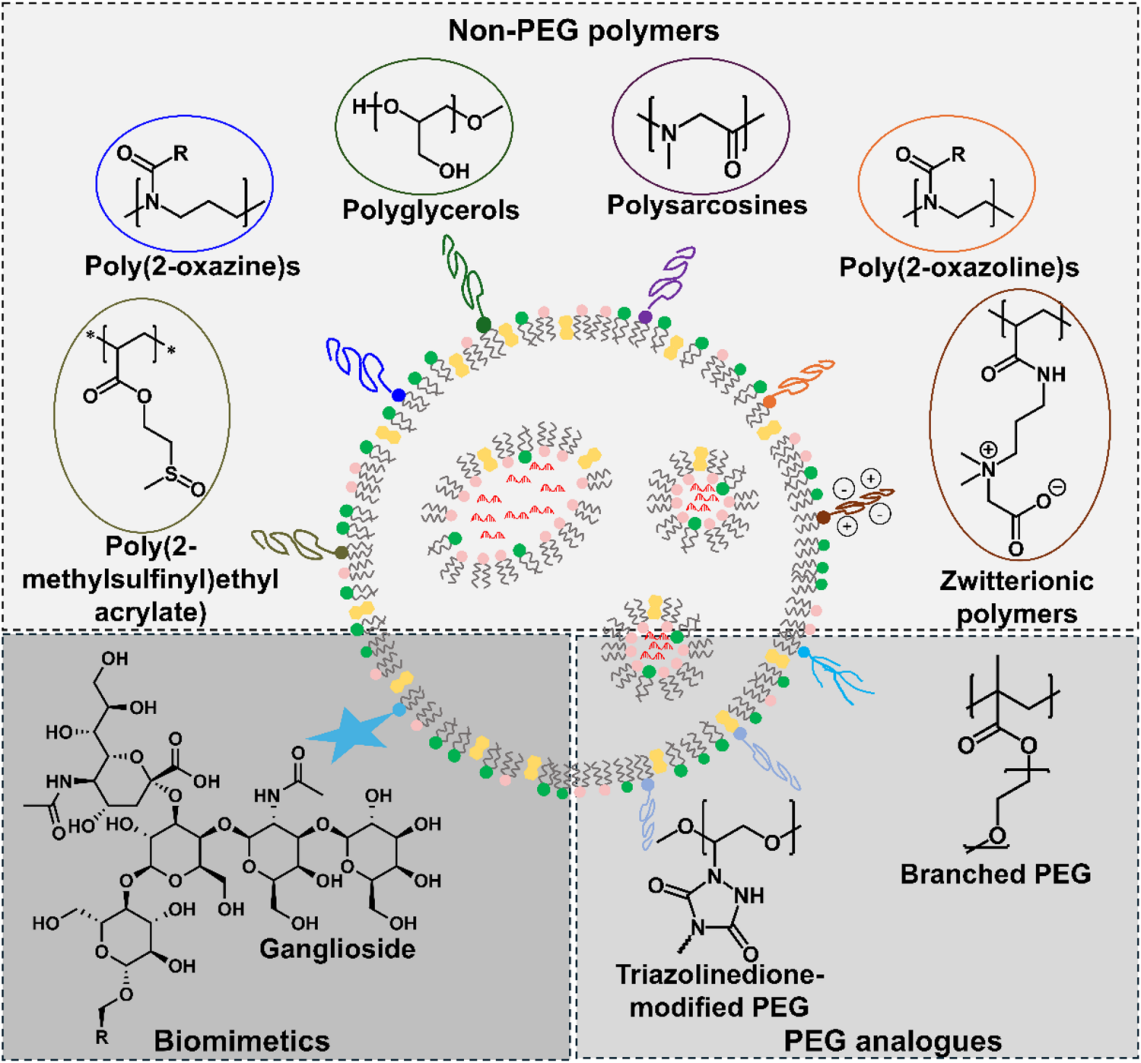
Representative structures of recent PEG‐lipid alternatives for LNP surface engineering, including non‐PEG polymers, PEG analogues, and biomimetic systems.

Several recent reviews have comprehensively summarized the design principles, clinical performance, and biological barriers associated with PEGylated LNPs for nucleic acid delivery [6, 7, 8, 9]. However, these studies primarily focus on conventional PEGylated LNP systems rather than on PEG‐lipid alternatives specifically designed for LNP surface engineering. Moreover, the rapid emergence of PEG‐associated limitations has shifted attention toward alternative stealth strategies. Recent commentary articles have further highlighted the growing importance of PEG alternatives and have focused on selected strategies designed to reduce anti‐PEG antibody responses, but do not systematically compare the broader range of PEG‐lipid alternatives for mRNA‐LNPs [10]. In parallel, broader reviews on stealth polymer alternatives have provided valuable overviews of PEG substitutes in biomedical applications. However, these discussions largely focus on polymer chemistry across different nanoparticle systems, rather than addressing how these polymers are lipid‐conjugated, incorporated into LNPs, and evaluated for LNP‐specific performance [11]. Here, we specifically highlight recent advances in PEG‐lipid alternatives for LNP surface engineering, focusing on non‐PEG hydrophilic polymers, e.g., polysarcosine (pSar), poly(2‐oxazoline)/poly(2‐oxazine) (POx/POz), poly(N‐vinyl amide)s, and polyglycerols (PG), and zwitterionic polymers (e.g., poly(2‐methacryloyloxyethyl phosphorylcholine) (PMPC) and poly(carboxybetaine) (PCB)), biomimetic and peptide‐based coatings (Proline/Alanine‐rich Sequence (PAS) peptides, albumin‐binding lipids, and gangliosides), and structurally modified PEG analogues (branched, cleavable, fluorinated, and brush PEGs) (Figure 1). This review is not intended to present a fully established structure–function framework, as the field of PEG‐alternative lipid for LNPs remains relatively early and the available studies are still limited, heterogeneous, and often not directly comparable.

Based on current knowledge, we critically evaluate how surface engineering strategies influence LNP physicochemical properties, biodistribution, intracellular trafficking, and immunological responses; and identify the key limitations that currently hinder rational for LNP surface design. These include LNPs stability under biological environments, limited repeated‐dose evaluation, incomplete immunogenicity analysis, insufficient understanding of polymer‐dependent protein corona dynamics, restricted architectural diversity beyond conventional end‐chain lipid conjugates, and the lack of standardized in vitro–in vivo evaluation frameworks. Because direct structure–function relationships remain incompletely defined, we outline strategic priorities and systematic structure–function studies to accelerate the rational design of stealth LNPs suitable for repeated and chronic therapeutic use, providing a translational roadmap for next‐generation LNPs.

## 2 Challenges With PEG‐Lipids in LNPs

**FIGURE 2:**
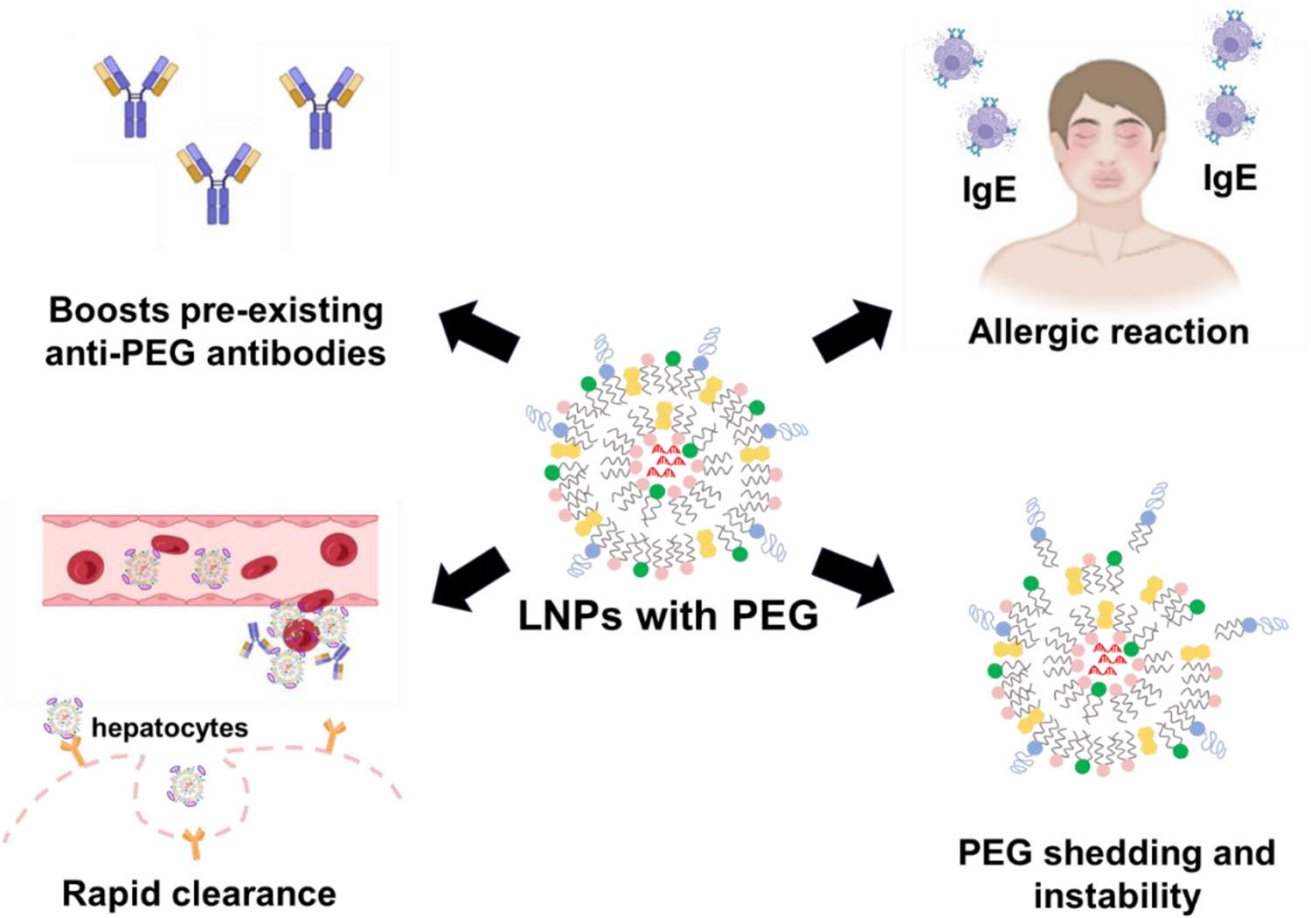
PEG provides steric stabilization and reduced opsonization but introduces critical drawbacks, including PEG shedding–dependent instability, hypersensitivity reactions, accelerated blood clearance (ABC), and widespread pre‐existing anti‐PEG antibodies. These limitations complicate repeated or chronic dosing and motivate the development of alternative stealth polymers. Some of the images were created with BioRender.com.

PEG has long been the dominant surface‐shielding polymer used in LNP formulations owing to its ability to provide steric stabilization and inhibit aggregation [12, 13, 14]. PEGylated lipids have been essential in enabling the clinical translation of mRNA–LNP therapeutics, most notably the first mRNA vaccines. However, PEG is increasingly recognized to present limitations that might challenge its continued use in next‐generation nanomedicines (Figure 2) [15]. Several excellent reviews have discussed these drawbacks and their consequences for nanomedicine applications recently [16, 17]. Notably, epidemiological studies suggest that a substantial fraction of the population (up to ∼40%) may harbor pre‐existing anti‐PEG antibodies [18, 19, 20]. It is assumed that extensive exposure to PEG‐containing cosmetics and consumer products has led to a rising prevalence of anti‐PEG IgM and IgG antibodies in the population [4, 14, 21, 22, 23]. These antibodies can accelerate blood clearance of PEG‐containing nanoparticles by altering biomolecular corona formation, a phenomenon known as the “ABC” effect, and diminish therapeutic benefit. This concern has become particularly relevant following the clinical use of PEGylated mRNA‐LNP vaccines. Anti‐PEG antibody levels were reported to increase after vaccination with Comirnaty (Pfizer‐BioNTech) and Spikevax (Moderna), with IgG increasing by 1.8‐fold and 13.1‐fold, and IgM increasing by 2.6‐fold and 68.5‐fold, respectively [4, 24]. Previous studies have shown that anti‐PEG antibodies can recognize PEG through non‐covalent interactions, including van der Waals forces and hydrogen bonding [25]. Current evidence does not yet clearly establish how elevated anti‐PEG antibody levels translate into clinical risk [5]. The biological processes that drive their formation in humans also remain insufficiently understood.

PEG immunogenicity is not determined by PEG alone, but also by how PEG is chemically presented in the nanoparticles. For example, hydrophobic moieties attached to PEG, including lipid anchors, may stabilize interactions with anti‐PEG antibodies and promote a more immunogenic PEG presentation [26]. Consistent with this, pure PEG nanoparticles lacking hydrophobic terminal groups show reduced recognition by pre‐existing anti‐PEG antibodies and limited anti‐PEG antibody induction in mice [27]. These findings indicate that PEG‐lipid associated limitations are possibly highly structure‐dependent and are influenced by PEG terminal chemistry, chain length, architecture, molecular weight, lipid anchor structure, and linker chemistry. These parameters may affect anti‐PEG antibody recognition, immune activation, and overall LNP delivery performances.

PEG‐lipid not only plays a critical role in modulating the pharmacokinetics and biological interactions of LNPs, but it also introduces important design trade‐offs [9]. One key factor is its tendency to undergo “shedding,” whereby PEG‐lipids dissociate from the LNP surface during systemic circulation. This process is primarily governed by lipid anchor chemistry and acyl chain length, and it significantly influences particle stability, circulation lifetime, immune recognition, and delivery efficiency [28, 29]. For example, during the development of ONPATTRO (patisiran), the use of rapidly shedding PEG‐lipids with shorter C14 anchors (1,2‐dimyristoyl‐rac‐glycerol (DMG)) was found to enhance blood clearance for hepatocyte uptake following intravenous administration [3]. In contrast, PEG‐lipids with longer C18 anchors (1,2‐distearoyl‐*sn*‐glycero‐3‐phosphorylethanolamine (DSPE)‐PEG) exhibit stronger membrane association, leading to reduced shedding and prolonged circulation half‐life, which can be advantageous for applications requiring sustained systemic exposure [3, 30]. These differences can affect protein corona formation, apolipoprotein (ApoE) recruitment, biodistribution, cellular uptake, and intracellular delivery. These observations highlight a fundamental trade‐off between circulation stability and biodistribution. This behavior reflects the broader “PEG dilemma” observed in nanomedicine, wherein PEGylation enhances nanoparticle stability and prolongs systemic circulation by reducing protein adsorption and immune recognition, but simultaneously impairs cellular uptake and intracellular delivery [31]. In LNP systems, this dilemma is closely tied to PEG‐lipid shedding dynamics. While stable PEG coatings improve pharmacokinetics, efficient delivery often requires partial or rapid PEG dissociation to expose the particle surface for cellular interactions. As such, balancing PEG retention and shedding remains a critical design challenge for optimizing LNP performance. The biological impact of PEG‐lipid shedding also depends on dose, dosing frequency, and administration route [32]. Higher or repeated doses may increase PEG‐lipid exposure and promote anti‐PEG antibody induction, complement activation, or accelerated blood clearance. Route of administration further changes the exposure environment, with intravenous delivery directly engaging circulating proteins and hepatic clearance pathways, while intramuscular or subcutaneous delivery involves local tissue retention and lymphatic drainage. Thus, PEG‐lipid shedding should be interpreted in dose‐ and route‐specific contexts.

Beyond anchor length, PEG architecture, including chain length, branching, and terminal group chemistry, also plays an important role in modulating complement activation and antibody responses, driving the development of branched and cleavable PEG designs to mitigate immunogenicity [33]. In addition, terminal functional groups enable ligand attachment for targeted delivery but may increase immune recognition or faster clearance if unmodified [34]. For targeted delivery, optimization of antibody ligand orientation and PEG length has been shown to improve targeting efficiency by preserving antibody accessibility and reducing interference from the biomolecular corona, thereby enhancing receptor binding in biological environments [24]. However, systematic studies examining how antibody/ligand size, density, and orientation influence LNP protein corona composition, reduced protein adsorption, prolonged circulation, targeting efficiency, and immune recognition remain limited. Importantly, most ligand‐functionalized PEG‐lipid strategies remain focused on distal end‐chain modification of PEG, rather than broader redesign of the PEG backbone or PEG‐lipid architecture. In particular, copolymerization‐based approaches, either combining PEG with other stealth polymers or introducing comonomers that modify hydration, flexibility, degradation, possibly targeting attachment, or immune recognition, remain largely unexplored in LNP surface engineering.

Although PEG has traditionally been regarded as biologically inert, increasing reports of hypersensitivity and anaphylactic reactions, particularly following administration of PEG‐containing vaccines, have raised safety concerns. These reactions are thought to arise, at least in part, from PEG‐induced complement activation leading to complement activation‐related pseudoallergy (CARPA) [18, 35].

**FIGURE 3:**
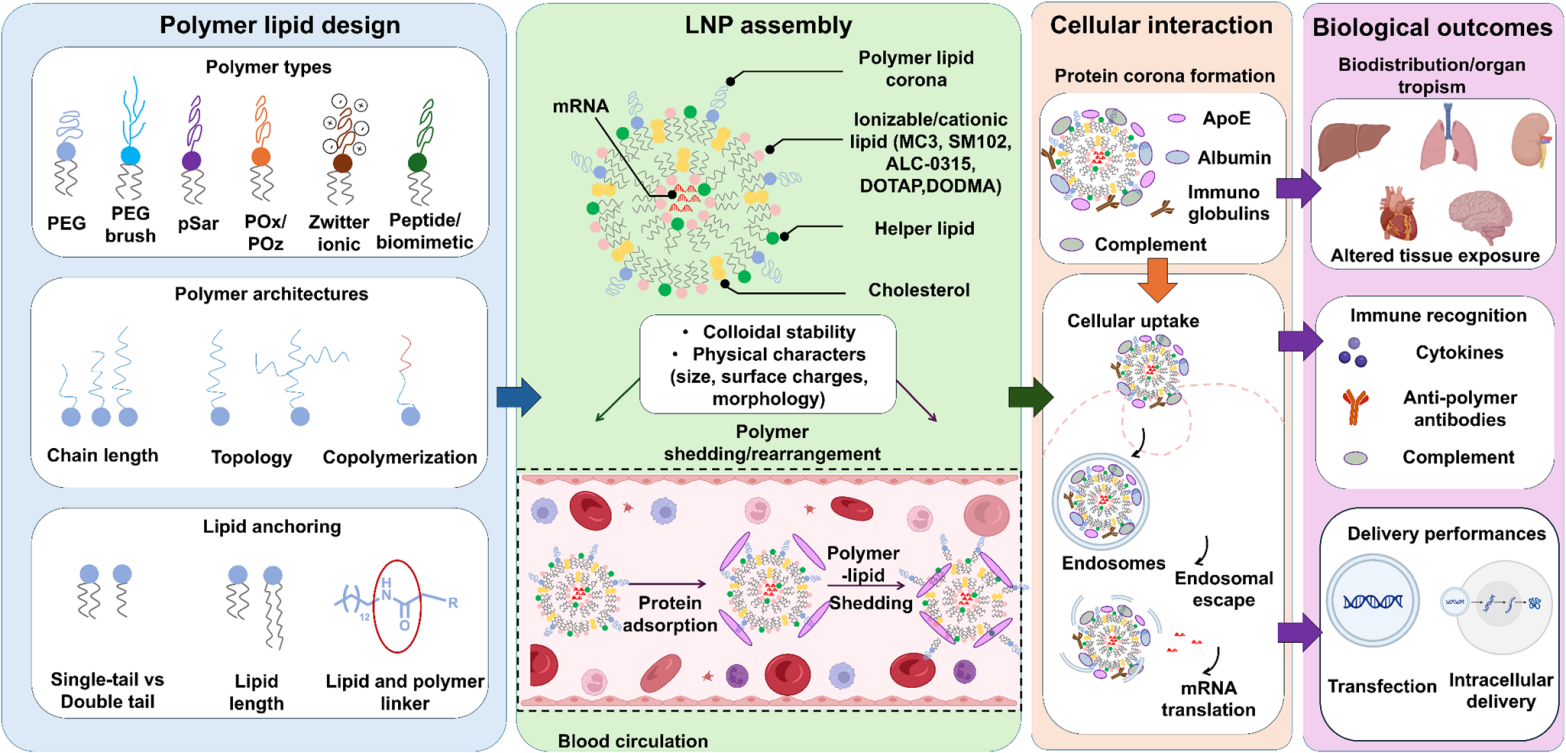
Conceptual design map linking polymer–lipid structure to LNP assembly, nano–bio interactions, and biological outcomes. Polymer–lipid design variables, including polymer type, polymer architecture, chain length, topology, copolymerization, lipid anchor structure, lipid length, and linker chemistry, influence LNP assembly, physicochemical properties, polymer shedding or rearrangement, and protein corona formation. These surface‐level changes can subsequently affect cellular uptake, endosomal escape, biodistribution, immune recognition, and intracellular delivery performance. Some of the images were created with BioRender.com.

As RNA therapeutics advance toward chronic, booster, and high‐frequency dosing applications, these drawbacks have spurred intensive research into alternative polymers that can provide stealth without the immunogenic liabilities of PEG. The same principle applies to the potential PEG‐alternative. As summarized in Figure 3, polymer identity, architecture, chain length, topology, copolymer composition, lipid anchoring, and linker chemistry collectively influence LNP assembly, colloidal stability, surface rearrangement, polymer shedding, protein corona formation, cellular uptake, endosomal escape, biodistribution, immune recognition, and delivery performance. This design map highlights that PEG lipid alternatives should not necessarily act as direct PEG mimics, but instead generate distinct nano–bio interfaces with material‐specific biological consequences.

## 3 PEG Lipid Alternatives for LNPs

To overcome the well‐recognized limitations associated with PEG [4], substantial research has been directed toward identifying and engineering new classes of hydrophilic, biocompatible polymers capable of providing stealth behaviour without the immunological liabilities associated with PEG for LNP surface modification. These next‐generation materials aim to retain the favourable hydration and steric stabilization offered by PEG while mitigating its drawbacks in vitro and in vivo [36].

Several classes of polymers have emerged as promising candidates for PEG for biomedical applications [11], such as zwitterionic polymers (e.g., PMPC, PCB, and poly(sulfobetaine)), hydrophilic polyacrylates, polyacrylamides, POx, POz, pSar, and polysaccharides including hyaluronic acid or dextran derivatives. These polymers bring a combination of tuneable architecture (e.g., chain length, branching, end groups), robust stealth behaviour, and reduced immunogenic risk. They represent a critical frontier for the next generation of LNP design, particularly for applications where repeated dosing or long‐term safety is essential. Their adoption has the potential to fundamentally improve nanoparticle circulation, reduce immune recognition, and enable safe and reproducible performance across repeated administrations, an essential requirement as mRNA vaccines, gene editing therapeutics, and nucleic acid drugs expand into broader clinical use cases.

In general, most PEG alternatives must likewise be chemically linked to lipid anchors prior to formulation. This modification enables their stable insertion into the LNP surface and allows the polymers to impart steric stabilization, hydration, and antifouling properties. In the following section, the major classes of lipid–polymer conjugates that have emerged as PEG‐lipid alternatives for LNP surface engineering are discussed individually, with emphasis on their distinctive physicochemical properties, antifouling behaviour, and in vivo performance relative to PEG. The different lipid‐polymers and their use in LNP formulations are summarised in comprehensive tables and selected examples for each lipid‐polymer are highlighted in more detail.

### 3.1 Polysarcosine (pSar)

**FIGURE 4:**
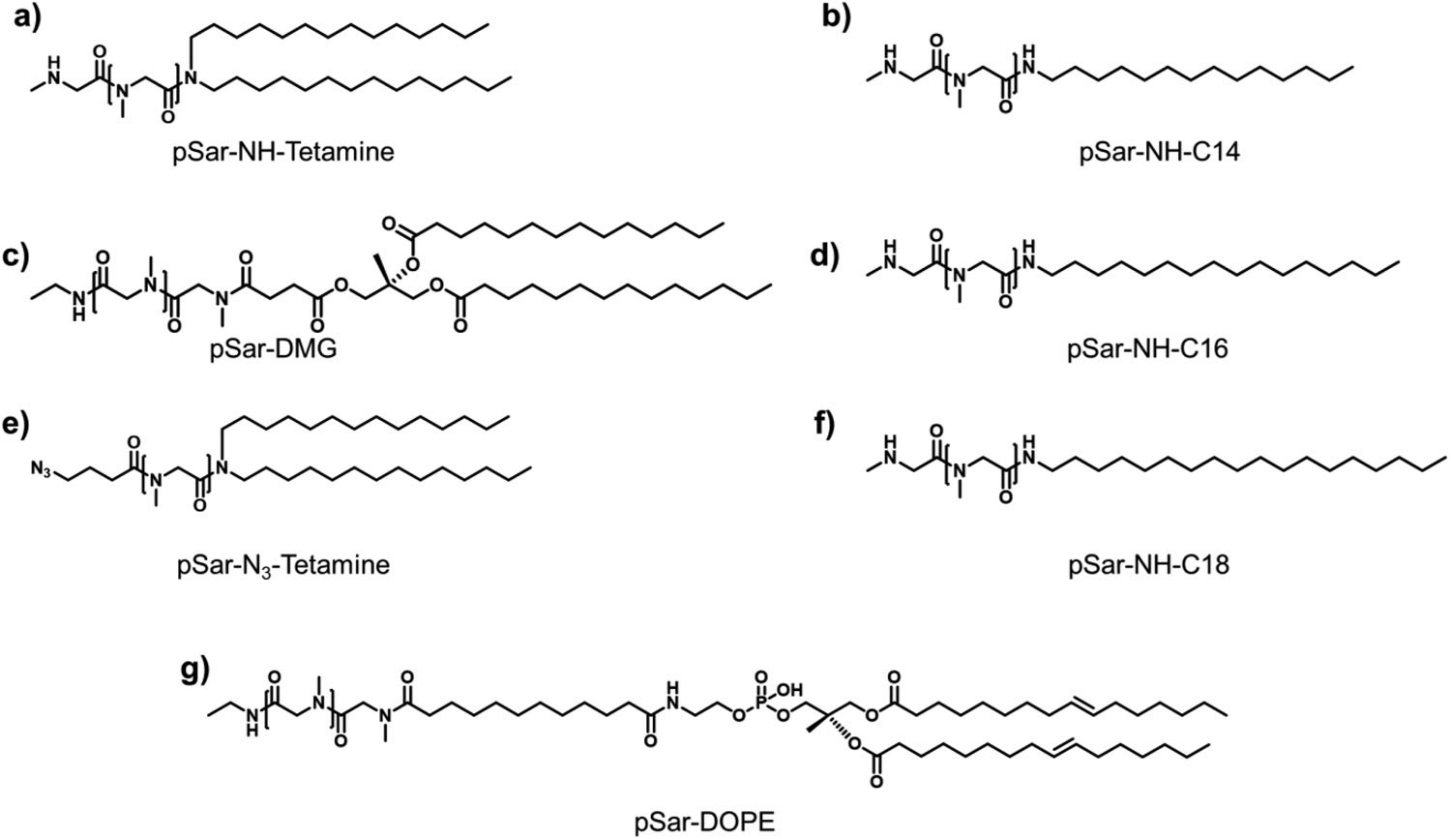
Schematic diagram illustrating the chemical structures of pSar–lipid conjugates. (a) pSar‐NH‐Tetamine [41], (b) pSar‐NH‐C14 [42], (c) pSar‐DMG [41], (d) pSar‐NH‐C16 [41], (e) pSar‐N_3_‐Tetamine [43], (f) pSar‐NH‐C18 [41], and (g) pSar‐DOPE [41].

pSar, a highly hydrophilic polypeptoid derived from N‐methylglycine, has become one of the most promising non‐ionic polymeric alternatives to PEG for LNP surface modification. Advances in controlled ring‐opening polymerization (ROP) of N‐carboxyanhydrides (NCAs) have enabled the synthesis of well‐defined pSar chains with controlled molecular weight, narrow dispersity, and tuneable end‐group functionality [37, 38]. As polypeptoids lack backbone hydrogen bonding, pSar adopts a flexible, highly hydrated conformation that resists protein adsorption. Compared with PEG, pSar offers similar water solubility but provides additional advantages including synthetic simplicity, chemical versatility, and biodegradability under acidic or enzymatic conditions with potential negligible immunogenicity [39, 40]. When conjugated to lipid anchors, pSar inserts stably into LNP membranes and forms a dense hydration layer as PEG. Figure 4 and Table 1 summarize the chemical structures of pSar‐modified lipids and their key physicochemical properties following formulation into LNPs.

**TABLE 1:** Summary of pSar lipid design and their key properties within LNP formulation.

No	Polymer	Lipid modification Strategy	Lipid length and type	Formulation	Typical structure / modification	Key properties: Size	Encapsulation Efficiency	Transfection Efficiency	Ref
1	pSar	Monoalkyl and bisalkylated amines as initiators	C14 and C18	DOPE:DOPC:Cholesterol:Polymer lipid	Linear, hydrophilic; pSar chain length of 44,	Comparable with PEG‐lipid	Comparable with PEG‐lipid	Higher in monocytes compared to PEG LNPs	[44]
2	pSar	Commercially available	C14 and C18	DOPE:DOTAP:Polymer lipid	Linear, hydrophilic with different DP of pSar	Comparable with PEG‐lipid	—	Increasing carbon diacyl chain length of pSar‐lipid led to lower expression in Hela cells and Jurkat T cells;	[43]
3	pSar		C14	DODMA:DSPC:Cholesterol:Polymer lipid	Linear, hydrophilic with different DP of pSar	Larger than PEG lipid	Comparable with PEG‐lipid	Lower than with PEG LNPs in HepG2 cells	[42]
4	pSar		C14‐pSar25, C16‐pSar25, C18‐pSar25, DOPE pSar25, SM102 pSar25,	ALC‐0315:DSPC:cholesterol:Polymer lipid	DP 25	The size doubles when the LNPs were formulated with either C16‐pSar25 or C18‐pSar25, and double lipid chained pSar lipids form comparable size with PEG LNPs	Comparable with PEG‐lipid	Single tail pSar lipid showed higher transfection compared to double tail pSar in comparison to PEG LNPs	[41]

Initial studies described the use of mono‐ and bis‐alkyl (C14/C18) initiators for the synthesis of pSar chains (degree of polymerization (DP) ≈ 44) through ring‐opening polymerization (ROP) [43, 44]. These pSar–lipids insert effectively into LNP bilayers with PEG‐like sizes and encapsulation efficiencies [44]. but displayed enhanced transfection in monocytes, suggesting that pSar's peptide‐mimetic backbone may modulate cell‐membrane interactions or endosomal trafficking in immune‐derived cells [44]. In related work, adjusting the acyl chain length altered intracellular activity. Longer hydrophobic anchors strengthened membrane insertion but reduced protein expression (4–6‐fold) in HeLa and Jurkat cells, indicating that overly strong anchoring may hinder membrane fusion or endosomal release [43].

**FIGURE 5:**
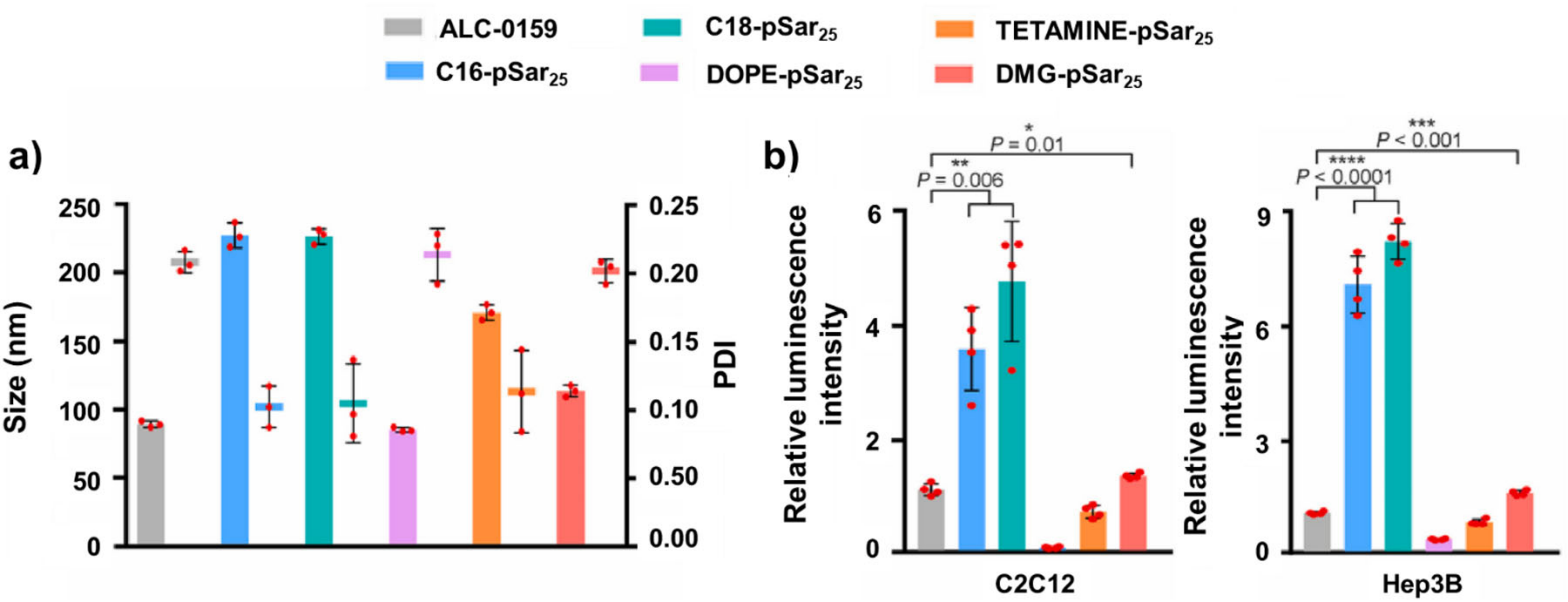
Particle properties of LNPs formulated with pSar lipids and mRNA delivery in cells. (a) Size, PDI, and encapsulation efficiency of ALC‐0315 based LNPs. (b) ALC‐0315 based LNPs relative luminescence intensity in C2C12 and Hep3B cells. Data in a and b, are presented as mean ± s.d. and statistical significance was analyzed by two‐tailed Student's *t*‐test. ^*^ *p* < 0.05, ^**^ *p* < 0.01, ^***^ *p* < 0.001, ^****^ *p* < 0.0001. Reproduced from [41] with Copyright 2024, Elsevier B.V. on behalf of KeAi Communications Co. Ltd. CC BY.

Chain‐length effects were further examined using C14‐pSar of varied degrees of polymerization, where increasing the proportion of pSar–lipid in the formulation unexpectedly improved transfection efficiency. This trend contrasts with PEG‐LNPs, where higher PEG density typically suppresses activity, and likely reflects the low steric hindrance imposed by pSar's soft, flexible corona. In vivo, pSar‐LNPs exhibited liver‐ and spleen‐dominant biodistribution similar to PEG‐LNPs, suggesting that pSar does not drastically alter systemic organ targeting [42]. A broader structural investigation comparing single‐tail and double‐tail pSar–lipids (C14, C16, C18, DOPE, DMG, tetamine; Figure 5) showed that single‐tail variants tend to form larger particles, often exceeding the size of PEG‐LNPs, yet achieve higher transfection efficiencies [41]. In contrast, double‐tail pSar–lipids produce sizes more closely aligned with PEG‐LNPs but exhibit somewhat reduced activity. These findings highlight the need for a finely tuned balance. Sufficient membrane anchoring is essential for stability [28], yet excessive rigidity may impede endosomal fusion and escape. Across studies, pSar‐modified LNPs demonstrate excellent safety, showing no elevations in liver enzymes and cytokine responses comparable to PEG‐LNPs.

Taken together, these results establish pSar as an adaptable and biologically compatible stealth material whose behavior diverges in important ways from traditional PEGylation. Rather than acting primarily as a steric barrier, pSar appears to create a softer, more dynamic hydration shell that preserves cellular engagement while still limiting immune recognition. Its peptide‐mimetic architecture enables interactions and intracellular trafficking pathways that differ from those of synthetic polyethers, which may explain pSar's enhanced performance in monocytes and its tolerance for higher surface densities without compromising activity.

### 3.2 Poly(2‐oxazolines) (POx) and Poly(2‐oxazines) (POz)

POx, including poly(2‐ethyl‐2‐oxazoline) (PEtOx), poly(2‐methyl‐2‐oxazoline) (PMeOx), and related POz such as poly(2‐methyl‐2‐oxazine) (PMeOz) and poly(2‐ethyl‐2‐oxazine) (PEtOz), have emerged as promising alternatives to PEG in LNP formulations. POx can be synthesized via living cationic ring‐opening polymerization (CROP) [45], enabling precise control over chain length, dispersity, and functional end groups. The tertiary amide backbone imparts strong hydrophilicity, tunable polarity, and inherently low protein adsorption. To date, different approaches have been presented for the design of lipid‐POx including direct termination [46, 47], and/or PPM conjugation strategies, including azide‐para‐fluoro thiol reactions [48], click chemistry [49], amidation [50, 51], and EDC/DMAP [51, 52] enable attachment to lipid anchors such as oleic acid, DMG [48, 53, 54], or C14/C18 monoalkyl chains [46, 55]. Another recent modular lipid‐POx platform was developed based on electron‐deficient alkyne lipids that allow conjugation to azide‐terminated polymers, including POx, via copper‐free azide‐alkyne cycloaddition [56]. These chemistries enable precise control over linker architecture, anchoring strength, and hydrophilic corona density, parameters known to influence LNP stability and bioactivity.

Efforts have been made to develop DMG‐like (double‐tail lipid) POx systems for LNP formulations. These formulation studies consistently show that POx‐modified LNPs exhibit particle sizes comparable to, or slightly larger than PEG‐based LNPs [53], with encapsulation efficiencies influenced by polymer DP and the choice of hydrophobic anchor [46, 48, 52]. Specifically, higher DP PEtOx can reduce cargo loading in certain formulations, while PMeOx‐based systems tend to retain high encapsulation efficiencies close to PEG benchmarks. Similar to pSar‐lipid, POx and POz‐lipids containing single lipid tails have been reported to form LNPs that are slightly larger (typically ranged 100–125 nm) compared to standard PEG LNPs [46]. Moreover, cyclic topologies of PMeOx have been shown to form LNPs with sizes in the range of 110–120 nm without compromising particle stability [51]. Transfection performance depends strongly on both polymer identity and anchor chemistry. Oleic acid–PEtOx LNPs show reduced activity compared to DMG‐PEtOx, although they still perform at levels comparable to PEG‐LNPs [48]. In contrast, PMeOx‐based LNPs exhibit robust transfection across multiple cell lines, including HEK293, DC2.4, 4T1, and IC21 [49]. Notably, cyclic PMeOx backbones result in lower cellular uptake and transfection efficiency than linear PMeOx and PEG‐LNPs across a wide range of cell lines, suggesting that cyclic PMeOx lipid grafts create a barrier to cellular interaction. However, cyclic polymers provide improved protein shielding and reduced protein binding, likely due to differences in corona architecture and membrane interactions [51]. Furthermore, POx and POz‐with single tail lipid‐based formulations exhibit cell‐type specificity, achieving high activity in RAW macrophages but reduced performance in Jurkat cells, highlighting the importance of structural fine‐tuning for targeted applications.

**FIGURE 6:**
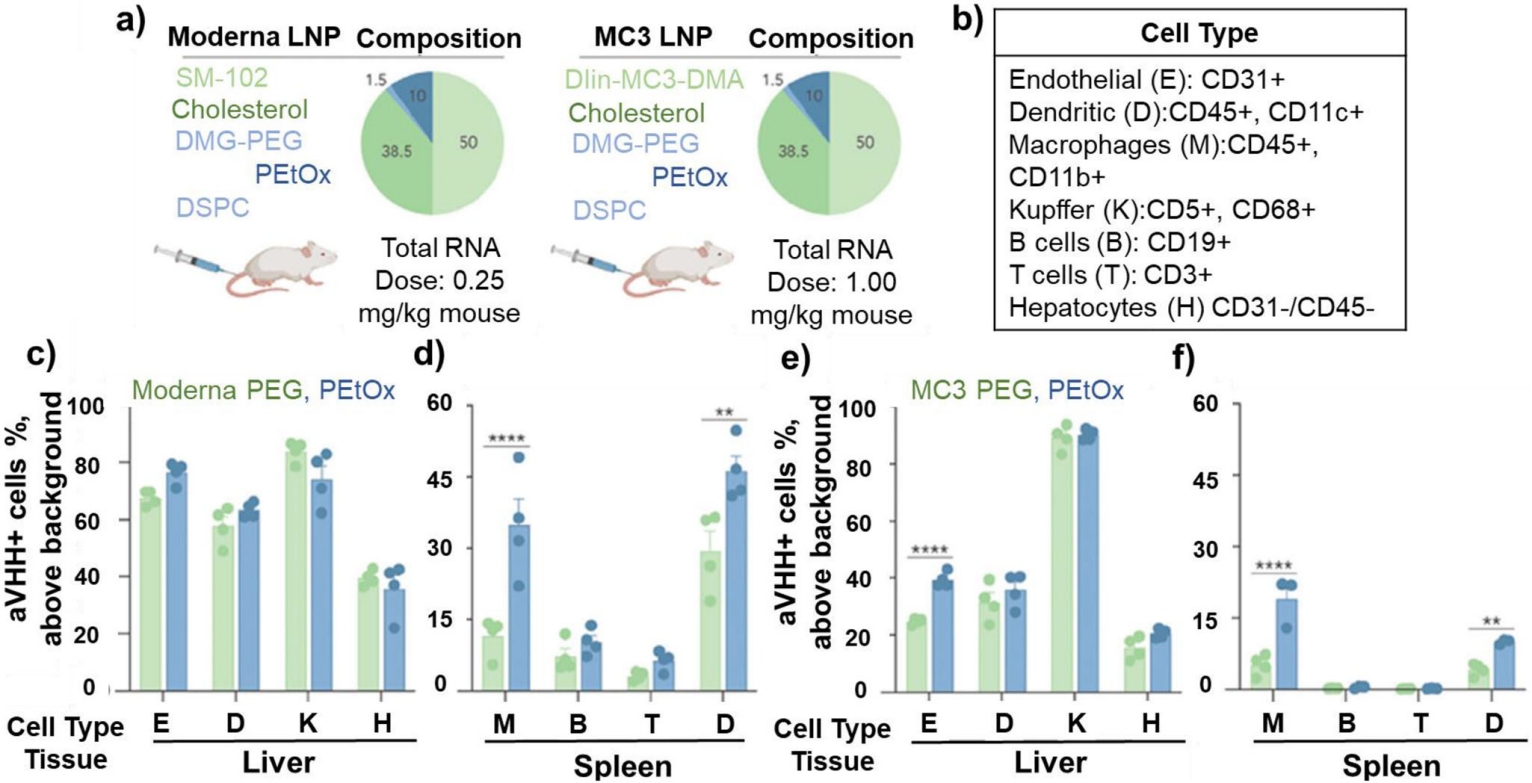
LNPs with PEtOx increase splenic antigen‐presenting cell (APC) delivery after intravenous administration. (a) Moderna and MC3 LNPs were formulated and administered intravenously at a dose of 0.25 and 1.0 mg kg^−1^, respectively. (b) Flow cytometry markers used in the analysis of cell types in the liver and spleen. (c) Moderna LNP formulated with PEtOx showed similar performance to Moderna formulated with PEG in liver cells (d), but significantly higher aVHH+ cells in spleen APCs. (e) Similarly, MC3 LNP formulated with PEtOx showed similar performance to MC3 formulated with PEG in liver cells (f) but significantly higher aVHH+ cells in spleen APCs. Statistical analyses were conducted using an ordinary two‐way ANOVA with Šidák's multiple comparison test between LNPs targeting the same cell type. Mean +/− S.E.M., ns (*p* > 0.05, not shown), ^**^ (*p* < 0.01), and ^****^ (*p* <0.0001). Reproduced from [53] with permission. Copyright 2024, John Wiley & Sons, Inc.

In vivo, PEtOx‐DMG‐LNPs (Figure 6) generally exhibit prolonged circulation and slower clearance than PEG‐LNPs after intravenous injection, most likely reflecting reduced opsonization and less immunogenic hydration shell [53]. Biodistribution of POx LNPs typically favours the liver and spleen but can extend to lung, kidney, heart, muscle, or lymph nodes depending on polymer chemistry and DP [48, 53]. Specifically, PEtOx‐containing Moderna LNPs exhibited significantly higher mRNA delivery to splenic macrophages and dendritic cells compared to PEGylated counterparts (Figure 6d). To determine whether this effect persisted across formulations, the study was repeated using MC3‐based LNPs (Figure 6e). While liver delivery remained similar across three of four cell populations, the enhanced delivery to splenic macrophages and dendritic cells was again observed for PEtOx‐containing LNPs (Figure 6f) [53]. In another study, short‐chain POx (DP ≈ 10) has been shown to increase liver and muscle accumulation relative to PEG‐LNPs, while certain POx derivatives reduce liver uptake overall [49]. Importantly, POx‐based LNPs exhibit favourable immunological profiles: PEtOx‐with para‐fluoro thiol linked‐DMG elicited both cellular immunity and antibody production without excessive acute toxicity [48]. PEtOx‐ether linked‐DMG (PEtOx‐O‐DMG) constructs induced lower levels of anti‐polymer lipid IgM with reduced opsonization and diminished accelerated blood clearance compared to PEG LNPs [53], and PMeOx‐LNPs generated strong neutralizing antibodies while producing lower inflammatory markers than PEG formulations [50]. Moreover, PEtOx‐LNPs, which showed minimal binding to anti‐PEG antibodies, offer a critical advantage for repeated dosing and long‐term therapeutic use [52].

Mechanistic analyses suggest that POx‐modified LNPs maintain endosomal escape efficiencies comparable to PEG systems, with outcomes most likely governed by the ionizable lipid rather than the hydrophilic polymer [51, 52]. Yet, subtle differences in corona hydration, polymer polarity, and membrane anchoring appear to modulate trafficking steps downstream of uptake, potentially explaining the reduced expression seen with cyclic PMeOx and the cell‐type‐specific effects observed across immune and non‐immune cell lines [51].

While numerous studies have explored POx‐lipid systems for LNP formulations and mRNA delivery, comparatively little attention has been given to POz. Kempe's group for the first time demonstrated that POz‐based LNPs exhibit physicochemical properties and in vitro and in vivo biological interactions comparable to those of similarly designed POx systems [46]. These promising results highlight the potential of POz to expand the design space for next‐generation LNP formulations.

Overall, POx‐based polymers represent a chemically diverse and highly tunable class of stealth materials whose behavior cannot be fully reduced to PEG mimicry. Their tertiary‐amide architecture enables precise modulation of polarity, rigidity, and hydration, offering comparable delivery performance as PEG. Going forward, the central challenge and opportunity lies in deciphering how POx structural variables (cyclic vs. linear topology, side‐chain polarity, DP, linker design, and anchor strength) collectively determine protein corona formation, intracellular trafficking, and immunological fate [57]. Establishing these structure–function relationships will not only optimize POx‐LNPs as PEG replacements but also position POx as a modular design platform capable of tailoring stealth behavior to distinct therapeutic contexts, including vaccines, gene therapies, and immunomodulatory applications.

**FIGURE 7:**
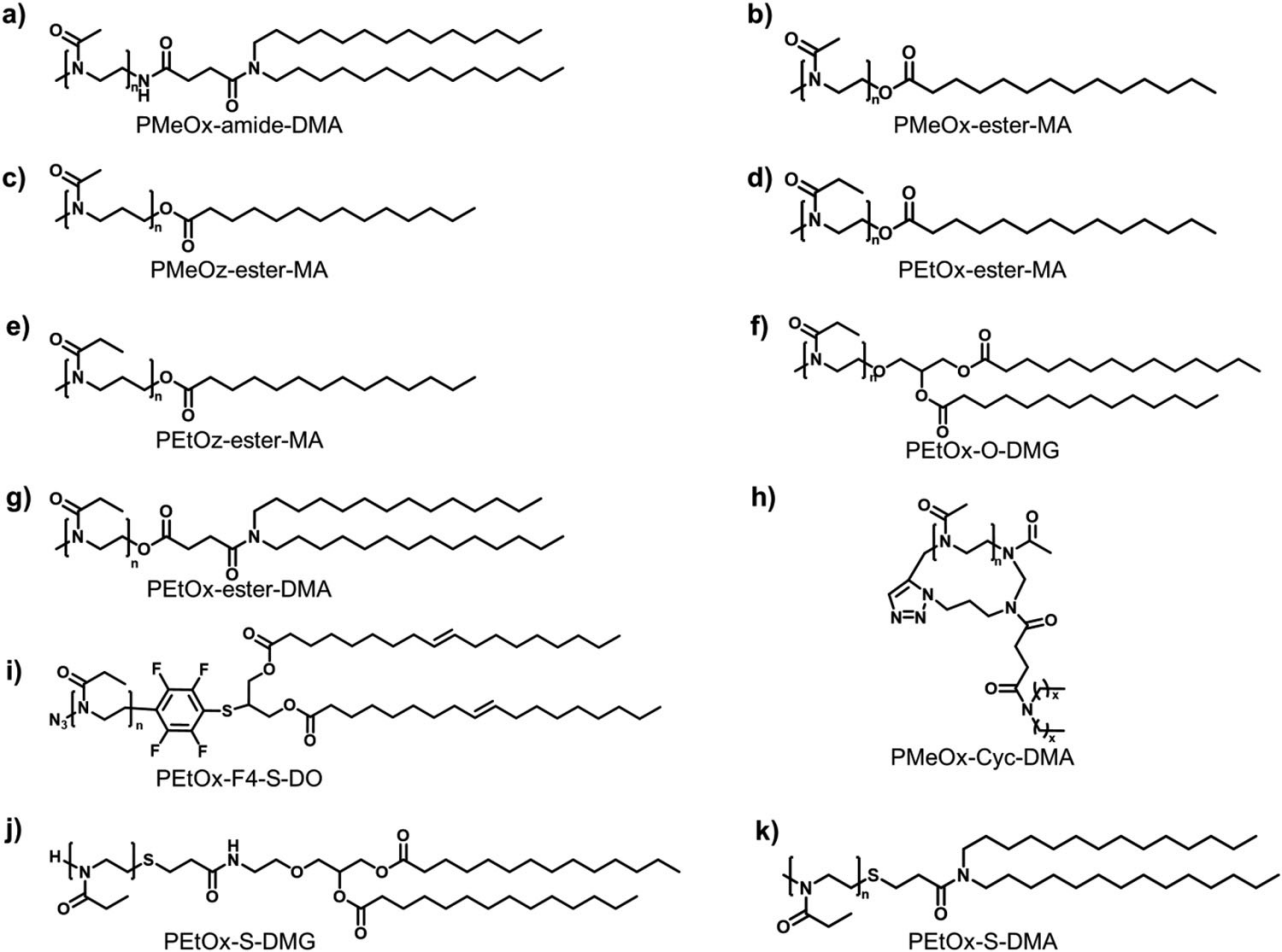
Schematic diagram illustrating the chemical structures of POx‐ and POz‐based lipid conjugates. *p* (a) PMeOx‐amide‐DMA [50, 51], (b) PMeOx‐ester‐MA [46], (c) PMeOz‐ester‐MA [46], (d) PEtOx‐ester‐MA [46], (e) PEtOz‐ester‐MA [46], (f) PEtOx‐O‐DMG [53], (g) PEtOx‐ester‐DMA [52], (h) PMeOx‐Cyc‐DMA [51], (i) PEtOx‐F4‐S‐DO [48], (j) PEtOx‐S‐DMG [54], and (k) PEtOx‐s‐DMA [54].

All POx–lipid structures that have been used for LNPs and their key properties are summarized in Figure 7 and Table 2.

**TABLE 2:** Summary of POx lipid design and their key properties within LNP formulation.

No	Polymer	Lipid modification Strategy	End‐group	Linker type (with lipid)	Lipid length and type	Formulation	Typical structure / modification	Key properties: Size	Encapsulation Efficiency	Transfection Efficiency	Circulation/Half‐Life	Ref
1	PEtOx	PPM	Azide	Para‐fluoro thiol	C18 (mono and di alkyl)	MC3:DSPC:Cholesterol:Polymer lipid	Linear, hydrophilic; DP of 44,	Comparable with PEG‐lipid	Comparable with PEG‐lipid	Single tail PEtOx LNPs lower than DMG‐PEtOx and DMG‐LNPs; comparable transfection of DMG‐POx and PEG LNPs in mice	—	[48]
2	PEtOx	provided by Serina Therapeutics	Methyl	Ether	DMG	MC3:DSPC:Cholesterol:Polymer lipid	Linear, hydrophilic; same DP	Bigger than PEG LNPs	Comparable with PEG‐lipid	—	POx LNPs show reduced clearance from the bloodstream than PEG LNPs.	[53]
3	Cyclic PMeOx	PMeOx with distearylamine derivatized containing succinic anhydride (DS‐COOH)		Amide	DSG:C18	ALC 0315 ionizable lipid:DSPC:Cholesterol:Polymer lipid	Linear and cyclic polymer	Comparable with PEG‐lipid	Comparable with PEG‐lipid	—	Protein bound reduced significantly in cyclic PMeOx compared to linear one, while linear PMeOx and PEG showed comparable protein binding	[51]
4	PMeOx	not stated	Methyl	Amide	DMG	ionizable lipid:DSPC:Cholesterol:Polymer lipid	Linear, hydrophilic; same DP	Comparable with PEG‐lipid	Comparable with PEG‐lipid	Comparable with PEG‐lipid in HeLa, 293T and HepG2 cells	—	[50]
5	PEtOx	EDC DMAP reaction with ditetradiacylamine	Methyl	Ester		ALC‐0315:DSPC:Cholesterol:Polymer lipid	Linear, hydrophilic; different DP	Comparable with PEG‐lipid	Lower than PEG LNPs;	Comparable with PEG‐lipid in HEK293T cells		[52]
6	PMeOxPEtOx PMeOzPEtOz	Termination of CROP of POx and POz	Methyl	Ester	C14 and C18 (Monoalkyl)	MC3:DSPC:Cholesterol:Polymer lipid	Linear, hydrophilic; same DP	Bigger than standard PEG LNPs	Comparable with PEG‐lipid	Comparable transfection in RAW cells and lower transfection in jurkat cells than standard PEG LNPs		[46]

### 3.3 Zwitterionic Polymers

Zwitterionic polymers have gained substantial attention as PEG alternatives for LNP surface engineering because their unique charge‐balanced repeat units create exceptionally strong hydration shells that prevent protein adsorption. Their internal pairing of positive and negative charges yields overall neutrality but high polarity, allowing them to mimic the zwitterionic character of natural phospholipids [58]. This combination results in remarkable antifouling behavior, resistance to protein adsorption [59, 60, 61], and responsiveness to pH or ionic strength [62, 63], which together support improved serum stability [64] during systemic delivery. As a result, zwitterionic polymers can interact minimally with pre‐existing anti‐PEG antibodies and provide a fundamentally different surface environment for LNPs than PEG–lipids [65, 66].

**FIGURE 8:**
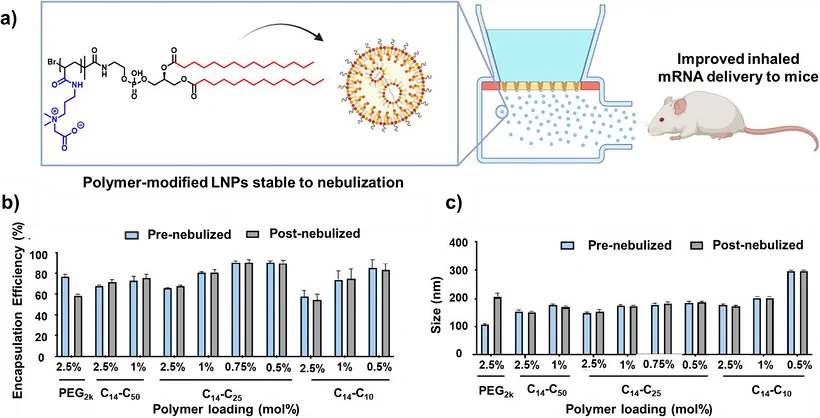
(a) DMPE‐PCB‐lipids for nebulized LNPs. (b) mRNA encapsulation efficiency and (c) size of PCB‐LNPs formulated with C12–200 ionizable lipid and C_14_ tail lipids of varying polymer length (*N* = 10, 25, 50) before and after nebulization. Reproduced from [70] with permission. Copyright 2024, American Chemical Society.

Within this class, phosphatidylcholine‐mimicking polymers such as PMPC have proven particularly effective. Because PMPC structurally resembles native membrane headgroups, it forms highly hydrated, sterically repulsive layers on particle surfaces. Early studies showed that PMPC‐containing amphiphilic copolymers integrate strongly into lipid membranes and markedly enhance stability in serum‐rich conditions [67]. Building on this foundation, atom‐transfer radical polymerization (ATRP)‐derived PMPC–lipids, generated using long‐chain lipid initiators such as distearoylglycerol, demonstrated that even low incorporation levels in liposomes dramatically reduce protein adsorption, with performance further boosted by increasing PMPC chain length [60]. Reversible addition–fragmentation chain transfer (RAFT) agent–functionalized lipid anchors of 1,2‐dipalmitoyl‐sn‐glycero‐3‐phosphoethanolamine (C16; DPPE) were used for the synthesis of DPPE‐PMPC. The resulting PMPC‐LNPs, though slightly larger than PEG‐LNPs, possessed comparable or improved mRNA expression depending on polymer length [68]. It was verified that they exhibited low cytotoxicity, minimal inflammatory activation in THP‐1 cells, and reduced in vivo cytokine responses relative to PEG‐LNPs, highlighting their excellent safety profile. Another zwitterionic polymer, PCB offers a distinct but equally powerful zwitterionic architecture [69]. Its strong hydration layer gives PCB potential lower immunogenicity than PEG. RAFT‐synthesized PCB–lipids using DMG or DSG anchors integrate seamlessly into standard LNP formulations [61]. These PCB‐decorated LNPs maintain sizes (85–150 nm) in the PEG‐LNP range while preserving high RNA encapsulation efficiency (65%–93%). Across diverse cell types, they consistently enhance transfection efficiency, reflecting improved cellular uptake and endosomal processing. In vivo, PCB‐LNPs overcome accelerated blood clearance, support efficient CAR‐T cell expansion, and exhibit minimal liver toxicity. ATRP‐prepared PCB–DMPE showed similarly promising behaviour, exhibiting physical properties comparable to PEG‐LNPs prior to nebulization [70]. Notably, these systems maintained particle size and encapsulation efficiency throughout the nebulization process, whereas PEG‐LNPs showed a marked reduction in encapsulation efficiency. Moreover, in vivo studies revealed a significant improvement in the inhaled delivery of mRNA to the lungs of both healthy and diseased mice compared to PEG‐LNP controls (Figure 8). In addition, PCB avoided the elevation of pro‐inflammatory cytokines during repeated dosing, demonstrating that its structure intrinsically supports both potency and tolerability.

A more recent zwitterionic candidate, pyridine carboxybetaine (PyCB), has been incorporated into LNPs by covalently attaching PyCB to the hydroxyl group of ALC‐0315, the ionizable lipid used in clinically approved mRNA vaccines [71]. This modification partially offsets decreased cholesterol content but also makes PyCB ratio a critical determinant of size and encapsulation efficiency. It was shown that high substitution can expand particle diameters up to ∼750 nm and compromise cargo loading if cholesterol or PEG are fully removed. Nevertheless, optimized PyCB‐LNPs exhibit reduced accelerated clearance, show PyCB‐dependent biodistribution across liver and spleen, and outperform PEG‐LNPs in suppressing tumor growth and metastasis. Importantly, they do so without elevating ALT/AST levels, confirming that the zwitterionic modification preserves hepatic safety despite structural changes to the ionizable lipid.

Taken together, these zwitterionic systems, whether phosphocholine‐mimetic, carboxybetaine‐based, or PyCB directly grafted onto ionizable lipids, demonstrate how careful structural tailoring can yield LNPs with strong antifouling behavior, controlled biodistribution, enhanced delivery efficiency, and reduced immune activation and toxicity. Their ability to combine stability with low immunogenicity makes zwitterionic–LNP systems a compelling structural platform for next‐generation nucleic acid therapeutics.

**FIGURE 9:**
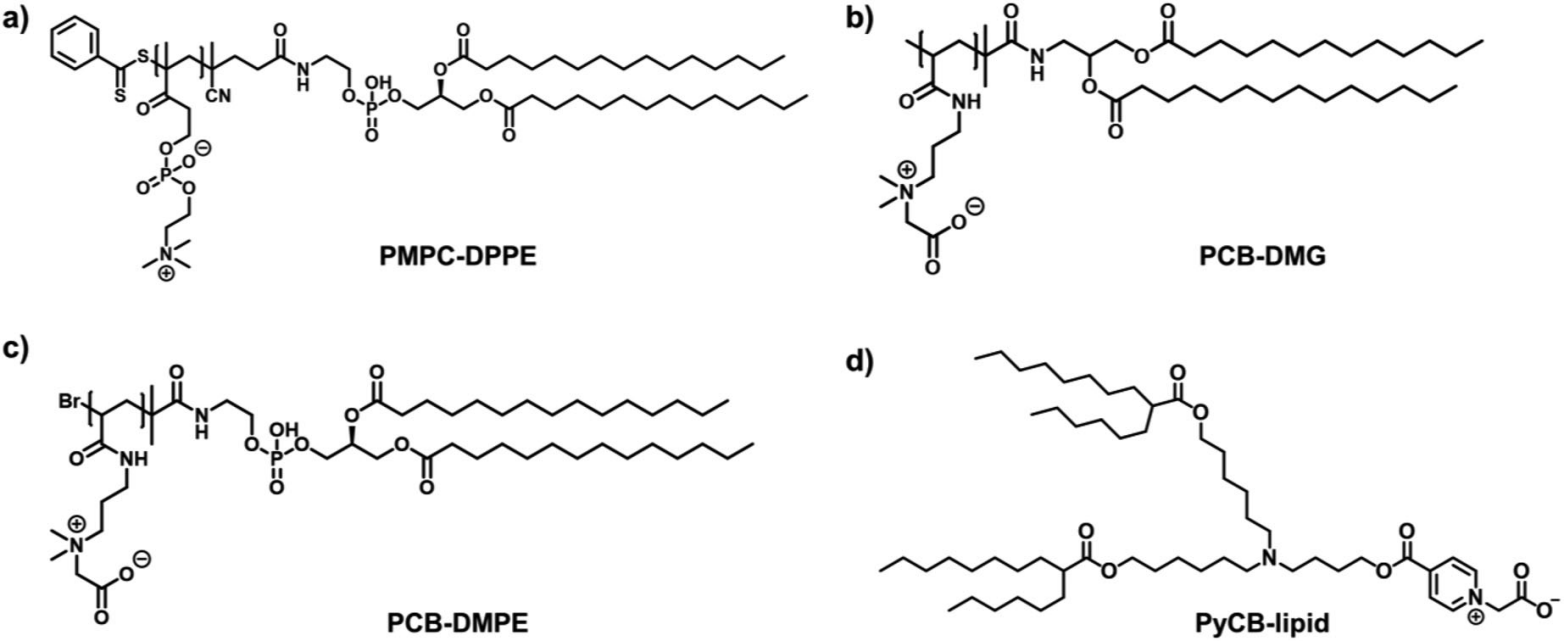
Schematic diagram illustrating the chemical structures of zwitterionic polymer–lipid conjugates. (a) PMPC‐DPPE [68], (b) PCB‐DMG [61], (c) PCB‐DMPE [70], and (d) PyCB‐lipid [71].

All zwitterionic polymer conjugated lipid structures that have been used for LNPs and their key properties are summarized in Figure 9 and Table 3.

**TABLE 3:** Summary of various zwitterionic polymer modified lipid design and their key properties within LNP formulation.

No	Polymer	Lipid modification Strategy	Lipid length and type	Typical structure / modification	Formulation	Key properties: Size	Encapsulation Efficiency	Transfection Efficiency	Ref
1	Poly(2‐methyacryloyloxyethyl phosphorylcholine)	RAFT agent modified lipid	Dialkyl C16: 1,2‐DPPE	Linear, hydrophilic	MC3:DSPC:Cholesterol:Polymer lipid	Bigger than PEG‐lipid; 140 nm‐250 nm depend on DP of polymer	Comparable with PEG‐lipid	Comparable with PEG‐lipid	[68]
2	PCB	RAFT agent modified lipid	DMG (C14) and DSG (C18)	Linear, hydrophilic	SM102:DSPC:Cholesterol:Polymer lipid	Comparable with PEG‐lipid; 85–150 nm depend on formulation composition	65%–93%	Higher transfection efficiency than PEG‐LNPs	[61]
3	zwitterionic pyridine carboxybetaine (PyCB)	covalent chemistry between a zwitterionic PyCB with the hydroxyl group in lipid	ALC‐0315	Headgroup's hydrophilicity	ALC‐0315 IL:PyCB IL:DSPC with or without Cholesterol	Increase PyCB concentration increase size (800 nm)	Full removal of cholesterol and PEGylated reduce the encapsulation significantly	Dependent on PyCB percentage	[71]
4	PCB	Functionalization of lipid with ATRP initiator	DMPE:C14	Linear, hydrophilic	IR 117‐17:DOTAP:Cholesterol:Polymer lipid	Comparable with PEG‐lipid	Comparable with PEG‐lipid	5‐fold higher compared to PEG LNPs in lung	[70]

### 3.4 PEG Structure Alterations

**FIGURE 10:**
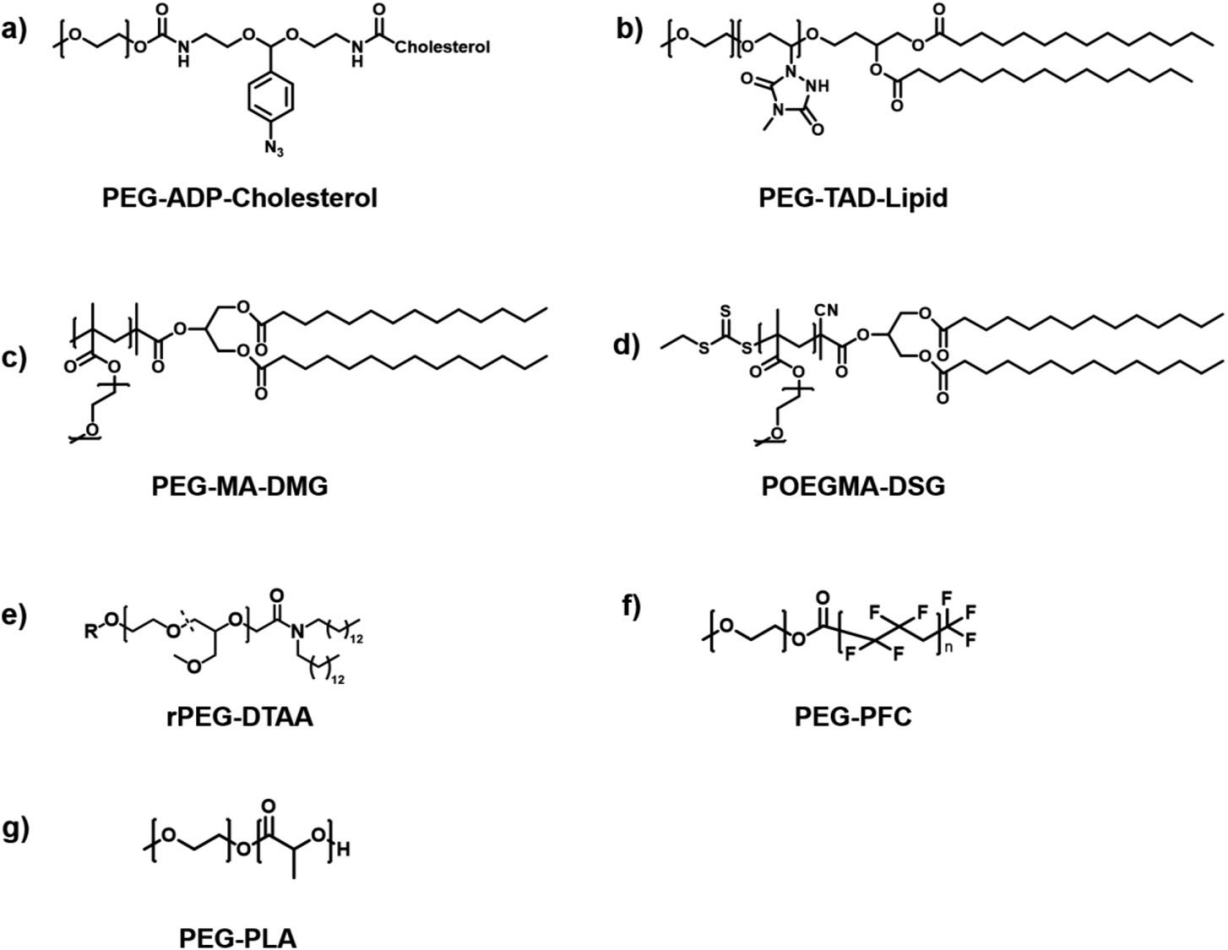
Schematic representation of PEG structural modifications designed to replace standard PEG–lipids in LNP formulations. (a) PEG‐ADP‐Cholesterol [72], (b) PEG‐TAD‐lipid [73] (c) PEG‐MA‐DMG [75], (d) POEGMA‐DSG [77], (e) rPEG‐DTAA [25], (f) PEG‐PFC [74], and (g) PEG‐PLA [78].

Rather than abandoning PEG entirely, several strategies have focused on structurally modifying PEG (Figure 10) to mitigate its limitations while preserving its favorable physicochemical properties. These approaches include PEG analogues incorporating degradable linkers, branched PEGs, isomerized PEG, and PEG modification with new functionalities [72, 73, 74, 75, 76, 77]. Introducing degradable linkers into PEG–lipid conjugates enables controlled de‐PEGylation in response to specific environmental triggers. For example, azido‐acetal, containing PEG–cholesterol conjugates [72] and triazolinedione (TAD)‐modified PEG lipids [73] undergo cleavage under pH‐ or temperature‐dependent conditions. This detachment exposes the nanoparticle surface after cellular uptake, thereby enhancing endosomal disruption and intracellular delivery. Importantly, these cleavable PEG coatings generally maintain particle size and encapsulation efficiency comparable to conventional PEG‐LNPs, yet significantly improve transfection efficiency, particularly in challenging tissues such as muscle and brain. Following intravenous administration, their biodistribution remains largely liver‐ and spleen‐dominant, consistent with standard PEG‐LNP behaviours.

Beyond cleavable PEG systems, architectural modifications to PEG further illustrate how polymer topology can profoundly influence LNP biological behaviour. Strategies such as brush‐type PEG architectures [75] and PEG isomerization [76] provide additional means to modulate their stealth performance without abandoning PEG chemistry altogether. Brush PEG–lipids, characterized by densely grafted PEG side chains along a polymer backbone with tunable length, were shown to form LNPs with particle sizes and encapsulation efficiencies comparable to conventional PEG‐LNPs but significantly enhanced circulation time and reduced anti‐PEG antibody binding [75, 77].

**FIGURE 11:**
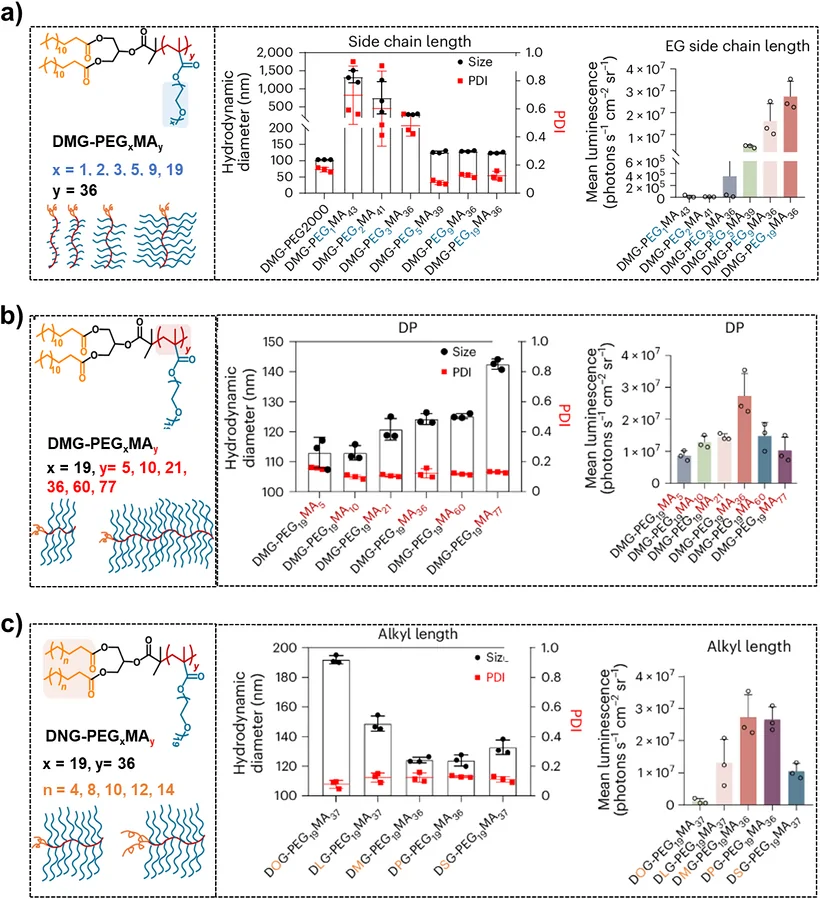
(a) Brush‐PEG‐lipid (BPL) chemical structure and schematic illustration with different EG side chain lengths. (b) BPL chemical structure and schematic illustration with different DP values, (c) BPL chemical structure and schematic illustration with different alkyl lengths. Size and polydispersity index (PDI) of BPL LNPs measured using dynamic light scattering. Data are presented as mean ± s.d. (*n* =  3 biologically independent samples). In vivo Luc mRNA delivery (0.1 mg kg^−1^) and d) Quantitative data of Luc activity in livers after Luc mRNA delivery. Data are presented as mean ± s.d. (*n* =  3 biologically independent samples). Data are presented as mean ± s.d. (*n* =  3 biologically independent samples). Reproduced and adapted from [75] with permission. Copyright 2025, Springer Nature Limited.

The Siegwart group synthesised a range of brush‐PEG‐lipid (BPL) by ATRP to generate a polymer lipid library with controlled architecture, chemical functionality and molecular weight using lipid‐functionalized initiators and various monomers containing linear, brush, hydrophilic, hydrophobic, positively charged, neutral and zwitterionic features. Their results demonstrated that increasing side‐chain length enhanced hydrophilicity, improved LNP formation, and reduced particle size to a stable ∼120 nm comparable to PEG‐LNPs (Figure 11) [75]. Optimal mRNA delivery was achieved at intermediate backbone length (DP ≈ 36), balancing stealth ability and cellular uptake, while both shorter and longer chains reduced efficacy. In addition, tuning lipid anchor length revealed that intermediate hydrophobicity (C10–C12) provided optimal stability and controlled desorption, resulting in superior in vivo transfection compared to shorter or longer anchors [75].

Isomerized PEG, also referred to as randomized PEG (rPEG), synthesized via anionic ring‐opening polymerization, represents another emerging approach to fine‐tune PEG architecture at the molecular level. By altering chain conformations and end‐group distributions [76], rPEG may offer distinct hydration and protein‐interaction profiles compared with conventional linear PEG. rPEG has recently been explored as a structurally modified PEG‐lipid for LNP formulations [25]. In this study, chain‐end functionalized rPEGs were conjugated with C14 lipid anchor groups of ditetradecylacetamide (DTAA), to generate rPEG‐DTAA. LNPs containing rPEG‐DTAA were then formulated and compared with PEG‐DTAA LNPs. These rPEG‐LNPs showed comparable sizes around 109–137 nm depend on degree of randomization with similar transfection efficiency across different cell lines. Importantly, anti‐PEG antibody recognition was negligible across all tested degrees of rPEG randomization. However, this antibody‐binding evaluation was not performed using complete LNP formulations, but rather using model lipid monolayers designed to mimic the LNP surface, consisting of 90 mol% 1,2‐dipalmitoylphosphatidylcholine (DPPC) and 10 mol% rPEG‐DMG. Therefore, while these findings suggest that PEG randomization can reduce anti‐PEG antibody recognition at the lipid interface, further studies are needed to confirm whether this effect is retained in fully formulated LNPs under biologically relevant conditions.

In parallel, alternative PEG modifications aim to introduce new chemical functionalities rather than cleavability. Notably, fluorinated PEG‐lipids have been developed to enhance hydrophobic interactions and modulate nanoparticle architecture [74]. Fluorination can reduce particle size and, when combined with ultrasound stimulation, enables stimuli‐responsive organ targeting, particularly to the spleen. These fluorinated PEG‐lipids promote enhanced endosomal escape and elicit stronger immune responses, including increased IFN‐γ–producing CD8^+^ T cells, compared with conventional PEG‐lipids. Such properties highlight their potential application in vaccine delivery and immunotherapy, where controlled immune activation is desirable. In addition, modification of linear PEG with biodegradable lipophilic poly(lactic acid) (PLA) structures can modulate tissue distribution and immune responses. PLA‐PEG slightly alters surface free energy and minimizes nonspecific protein interactions while maintaining transfection performance equivalent to standard LNPs [78].

Overall, these PEG modification strategies demonstrate that the limitations of PEG in LNP systems can be mitigated through rational chemical and architectural design rather than complete replacement. Cleavable linkers, functional substitutions, and topological tuning have shown potential to preserve PEG's favorable physicochemical properties while improving intracellular delivery. Importantly, these approaches highlight PEG topology and dynamics, not merely its presence, as critical determinants of biological performance. Such insights position structurally engineered PEG‐lipids as practical, translationally attractive solutions that bridge the gap between conventional PEGylation and next‐generation stealth strategies. From a regulatory perspective, PEGylated systems may benefit from a more established safety and clinical history, which can facilitate development and streamline aspects of FDA review compared to entirely novel excipients. In contrast, alternative polymers that have not been previously used in humans may face additional scrutiny, including more extensive preclinical safety, immunogenicity, and toxicology evaluations. Nevertheless, such strategies may only represent a temporary mitigation rather than a long‐term solution to PEG‐associated challenges. Even with modified PEG structures, repeated exposure could still boost anti‐PEG antibody responses over time, potentially broadening the antibody repertoire and exacerbating accelerated blood clearance. Importantly, most preclinical studies, particularly in murine models, do not capture these longer‐term immunological effects. As a result, the durability of these approaches in avoiding PEG‐related immunogenicity remains uncertain, and they may ultimately serve as interim solutions rather than definitive alternatives.

### 3.5 Other Alternatives

Several other alternative stealth polymer‐lipids and lipid‐modified materials have been explored to replace PEG‐lipid in LNP formulations, each offering distinct physicochemical, biological, and immunological advantages. While many of these alternative systems show promising initial results, most studies remain at an early stage, and some approaches present their own formulation or biological challenges that require further investigation.

#### 3.5.1 Poly(N‐vinyl amide)s

Poly(N‐vinyl amide)s stand out due to their hydrophilicity and biocompatibility. Poly(vinyl pyrrolidone) (PVP), the most extensively studied member, has been modified with lipid moieties for incorporation into liposomes and lipoplexes [78, 79, 80, 81]. For example, lipid chains were introduced via RAFT polymerization followed by amidation to produce DSPE‐PVP or mono‐ and dialkyl PVP lipids [81]. These PVP lipid modifications allow lipoplexes to maintain comparable size and encapsulation efficiency to PEG‐lipoplexes, with transfection outcomes influenced by both lipid architecture and polymer chain length [81]. In addition, DSPE‐PVP reduces IgM and IgG production and can efficiently prevent nanoparticle–plasma protein interactions, although some loss of in vitro efficacy remains, similar to PEGylated systems. While these studies demonstrate the promise of alternative hydrophilic polymers for nucleic acid delivery, their application in standard LNP formulations has remained largely unexplored. Building on this approach, poly(N‐methyl‐N‐vinylacetamide) (PNMVA), as a less explored poly(N‐vinyl amide)s class, has been explored for LNP surface coating. PNMVA was modified with DSPE through amidation, producing linear hydrophilic polymers with chain lengths of 24 or 50, which were then formulated into LNPs. PNMVA‐modified LNPs are slightly larger than DSPE‐PEG systems but retain encapsulation efficiency. They exhibit higher transfection efficiency in monocytes than PEG‐LNPs and show prolonged circulation with biodistribution across the kidney, heart, liver, lung, and spleen. Importantly, PNMVA LNPs do not compromise cellular metabolic activity and form a stable protein corona, demonstrating stealth characteristics similar to PEG‐LNPs without inducing ABC or hypersensitivity reactions, highlighting the potential of poly(N‐vinyl amide)s as PEG substitutes [82].

#### 3.5.2 Polyglycerols (PGs)

PG with their multivalent hydroxyl surfaces, provide strong hydration, low protein adsorption, and biocompatibility. Moreover, PG‐coated nanoparticles have shown reduced complement activation compared to PEG‐coated analogues, making them suitable for coating LNPs used in intravenous drug delivery [83, 84]. PG‐modified lipids have been synthesized via esterification of PG at DP 27 or 42 containing various functional groups with DMG and applied in LNP formulations. These PG‐DMG LNPs are generally larger than PEG‐LNPs, and their encapsulation efficiency depends strongly on the nature of the polyglycerol functionalities. Hydroxyl‐bearing polyglycerol LNPs perform similarly to PEG‐LNPs, while substitution with other functional groups reduces encapsulation efficiency. Transfection levels remain comparable to PEG‐LNPs, and these formulations exhibit negligible anti‐PEG antibody binding, suggesting a decreased likelihood of ABC effects upon repeat administration [85].

#### 3.5.3 Polysulfoxides

Recently, sulfoxide polymers, polymeric analogues of dimethyl sulfoxide (DMSO), a small molecule with an established safety profile, have emerged as promising PEG alternatives for biomedical applications due to their low‐fouling, biocompatible, and noninflammatory characteristics [86, 87, 88]. These findings highlight the strong potential of sulfoxide‐containing polymers as effective materials to replace PEG‐lipids in LNP formulations. Whittaker and team developed poly(2‐(methylsulfinyl)ethyl acrylate) (PMSEA) conjugated to DSPE via RAFT polymerization using a lipid chain transfer agent. Incorporation of PMSE‐DSPE in a standard LNP formulation resulted in slightly larger LNPs compared to PEG LNPs. Importantly, PMSEA LNPs showed more effective transfection with lower cell association levels, suggested to be due to a higher efficiency of endosomal escape in vitro [89].

#### 3.5.4 Polypeptides

Building on the success of pSar as a PEG alternative, recent advances have highlighted the broader feasibility of using polypeptides to replace PEG in nanoparticle systems. PASylation, which employs hydrophilic polypeptide chains composed of proline, alanine, and serine, has been shown to significantly extend the in vivo half‐life of protein therapeutics [90, 91, 92]. Inspired by this concept, polypeptide‐based lipids from the same PAS sequence have emerged as a refined strategy for LNP surface modification [91]. These PAS–lipids generate LNPs with particle size, RNA encapsulation, circulation behavior, and biodistribution profiles closely matching those of PEG‐LNPs, yet with notably reduced hepatic accumulation and an absence of anti‐PEG IgM induction. Together, these features underscore their improved immunological safety and demonstrate that polypeptide‐derived hydrophilic domains offer a structurally elegant and biologically compatible alternative to PEG. The main technical hurdles are scalable synthesis and potential protease susceptibility in vivo. Future work should explore peptidomimetic substitutions to improve metabolic stability, optimize lipid tail geometry for membrane insertion, and probe how sequence motifs influence corona composition and cellular trafficking.

#### 3.5.5 Ganglioside

Instead of modifying PEG or replacing it entirely with a new polymer, LNPs have also been developed incorporating ganglioside, a naturally occurring stealth lipid, as a PEG‐free alternative. Ganglioside is an amphiphilic molecule with a hydrophobic ceramide tail and a hydrophilic glycan headgroup linked through a glycosidic linkage [93]. Its hydrophobic tail allows van der Waals interactions with other lipids, stabilizing ganglioside within lipid membranes, while its hydrophilic glycan chain extends outward, exposing the sialic acid moiety that mediates cell communication [93]. The particles obtained are slightly larger than PEG‐LNPs but they retain encapsulation efficiency, improve transfection in HEK293T cells, and show biodistribution patterns similar to PEG‐LNPs, illustrating the promise of biomimetic lipids [94]. The main challenges are particle enlargement and the complexities introduced by glycan chemistry; heterogeneity of natural gangliosides, potential receptor‐mediated uptake differences across tissues, and batch‐to‐batch variability. Advancing ganglioside systems will require careful definition of glycan composition (synthetic versus natural), control over ceramide tail saturation/length to optimize membrane packing, and targeted biodistribution studies to map receptor interactions across cell types.

#### 3.5.6 Albumin‐Binder–Modified Lipids (AB‐Lipids)

**FIGURE 12:**
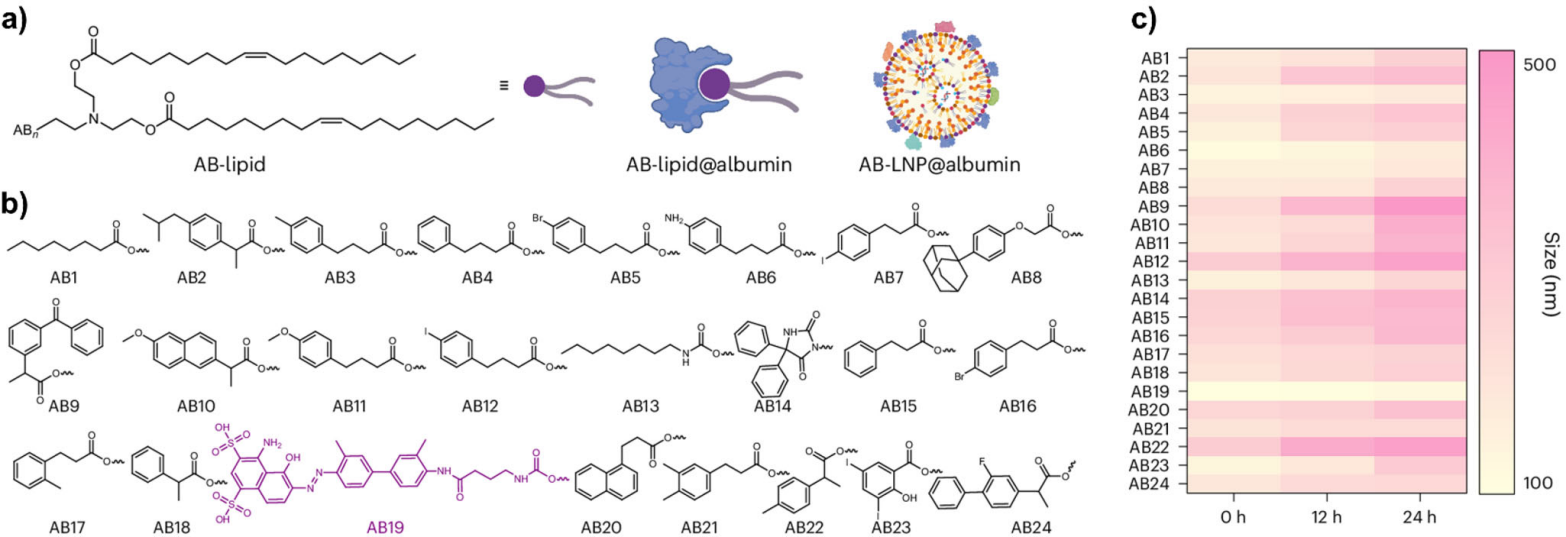
(a) Linkage strategy of AB‐lipid and the molecular complex formed between AB‐lipid and albumin. AB‐lipid was utilized for the preparation of AB‐LNP by a microfluidic chip. (b) Chemical structures of AB‐lipid library (AB1–AB24). (c) Changes in the average diameter of AB‐LNP over time determined by dynamic light scattering. Reproduced from [95] with permission. Copyright 2025, Springer Nature Limited.

Beyond polymer‐based coatings, other strategies exploit naturally occurring or biologically inspired materials. Albumin‐binder–modified lipids (AB‐lipids) represent a promising strategy, where dioleate‐tail ionizable lipids are conjugated to albumin binders through ester or amide linkages [95]. A library of ionizable lipids functionalized with classical albumin ligands (Figure 12), designated as ABs, was prepared. These molecules exhibited strong affinity for albumin [95]. The resulting AB‐LNPs had average sizes of 90–500 nm and maintained encapsulation efficiencies comparable to PEG‐LNPs. In particular, the AB19 lipid showed the most promising performance, forming ∼110 nm particles when encapsulating mRNA and demonstrating high stability, suggesting its potential as a long‐term alternative to traditional PEG lipids. In vivo, AB19‐lipid LNPs achieve higher expression levels than PEG systems and demonstrate distinct biodistribution following intramuscular delivery, distributing to muscle, heart, liver, spleen, lung, and kidney. Compared with PEG formulations, AB19‐lipid LNPs accumulate less in the liver and spleen and show excellent antitumor and antiviral therapeutic performance, eliciting strong cellular and humoral immune responses. Remarkably, these LNPs do not trigger specific antibody production or liver toxicity, maintaining normal biochemical markers and showing no pathological damage.

Taken together, the studies reviewed here show that each of the alternative structures examined brings a distinct combination of advantages and residual challenges that merit targeted optimization rather than complete replacement of existing formulations.

## 4 Perspectives, Current Limitations and Opportunities

### 4.1 Key Design Principles and Considerations

These diverse approaches illustrate the growing toolkit of PEG lipid alternatives, ranging from synthetic polymers to peptide‐based and biomimetic strategies. Each offers unique advantages and challenges, and their development highlights the continuing evolution of LNP design toward safer, more effective, and immunologically compatible delivery systems. Although no single material yet surpasses PEG across all metrics, the collective progress demonstrates that PEGylation is no longer the only viable option. Instead, a new generation of adaptive, modular stealth chemistries is emerging, capable of improving repeat‐dose tolerability, minimizing unwanted immune responses, and enabling application‐specific optimization. Continued development will likely shift the field toward tailored stealth strategies that integrate polymer, lipid, and biologically inspired components, paving the way for safer and more effective next‐generation LNP technologies. The availability of PEG‐lipid alternatives will further allow for a personalized medicine approach if patients show adverse reactions to one of the other. Thus, it is essential that the field further matures so that the full potential of each alternative system can be revealed and applied. Although the available studies do not yet support definitive universal design principles for PEG‐lipid alternatives, several emerging structure–performance considerations can be identified.

#### 4.1.1 General “PEG‐Lipid Substitution” Considerations

Most reported PEG‐lipid alternatives for LNP surface modification have been implemented as polymer chains bearing lipid conjugates at the chain terminus. Typically, a hydrophobic anchor (single‐ or double‐tail lipid) is used to tether the polymer onto the LNP surface [46, 61, 68]. This “lipid modified end‐chain” approach is attractive because it is straightforward to synthesize, integrates seamlessly into existing LNP manufacturing workflows, and reliably drives surface presentation of the polymer corona. However, it also imposes a constrained set of structural options (anchor length, single vs. double tail, and polymer DP). As a result, most studies have optimized PEG lipid alternatives by matching conventional PEG‐lipid parameters rather than exploring fundamentally new polymer–lipid geometries. Future studies should therefore move beyond simply replacing PEG within conventional PEG‐lipid design rules and systematically explore broader polymer–lipid architectures to define how surface‐retention profiles, shedding behavior, protein corona formation, and delivery performance are controlled.

#### 4.1.2 Polymer‐Lipid Composition Considerations

End‐chain attachment fixes the polymer's point of insertion and largely determines its mobility, surface density, and shedding kinetics, all parameters that control corona thickness, packing, and exposure of the underlying ionizable lipid. As a result, two polymers with similar chemistry can produce very different biological outcomes simply because of differences in anchor length, chain architecture, or lipid packing. Because most studies have focused on end‐chain conjugates, important regions of the design space remain underexplored. First examples have emerged as detailed in Section 3 of this review, but further advancements are needed to reveal the full potential of polymers. Each of the alternative design strategies would change polymer conformation, shedding behavior, and the dynamic reorganization of the LNP at the cell membrane. Exploring these alternative architectures is likely to reveal new structure–biology relationships that cannot be predicted from end‐chain conjugation strategies alone. For example, attaching lipids along the polymer backbone may alter endosomal escape efficiency or serum protein binding in ways that conventional single‐ or double‐tail lipid anchors cannot achieve. In addition, graft‐from approaches could enable significantly higher local grafting densities without the steric crowding typically associated with end‐functionalized systems. To move beyond empirical optimization, more systematic studies are needed that vary anchor chemistry, attachment point, polymer topology (e.g., linear, branched, bottlebrush), and biodegradability, and then correlate those parameters with protein corona composition, in vivo stability, cell‐type uptake, endosomal release, and immunogenic readouts. This need is illustrated by PEG alteration strategies discussed above, including brush‐like and isomerized PEG‐lipids, where changes in polymer architecture can influence circulation behavior and anti‐PEG antibody recognition [25, 75, 77]. However, these examples remain limited to specific formulations and do not yet establish general design rules.

#### 4.1.3 Polymer Chain Length and Grafting Density Considerations

PEG lipid alternatives are often synthesized with DP ∼40–45 to approximate the molecular weight of PEG2000, or with DP ∼20–25 to resemble shorter PEG chains. Similarly, lipid anchor design has largely followed established PEG‐lipid structures, commonly using double‐tailed C14 or C18 anchors because of their known effects on PEG‐lipid retention and shedding. Although these designs provide useful PEG‐like comparators, they also narrow the accessible design space. Polymer chain length and grafting density are central parameters because they influence corona thickness, surface coverage, LNP size, colloidal stability, and exposure of the underlying lipid surface. Longer or more densely grafted hydrophilic chains may improve steric stabilization and reduce nonspecific protein adsorption; however, excessive shielding may also suppress cellular uptake, membrane fusion, or endosomal escape. Conversely, insufficient surface coverage may expose the LNP core to serum proteins and immune cells, leading to opsonization, rapid clearance, or altered biodistribution.

#### 4.1.4 Polymer Topology Considerations

Linear, cyclic, branched, and bottlebrush architectures may form distinct hydration layers, chain conformations, and surface packing arrangements. These differences can influence not only the overall extent of protein adsorption, but also the composition of the protein corona, including the recruitment or depletion of ApoE, albumin, immunoglobulins, complement factors, and other serum proteins. Because protein corona composition can regulate organ tropism, cellular uptake pathways, immune recognition, and clearance, polymer topology should be considered an important design parameter in future PEG‐alternative LNP studies. Among hydrophilic polymer–lipid systems, only limited studies have directly compared different polymer topologies within the same polymer class. The most prominent one compares the performance of linear and cyclic PMeOx lipids in LNP formulations [51]. Cyclic PMeOx‐based LNPs showed lower cellular uptake and transfection efficiency than linear PMeOx‐ and PEG‐based LNPs, potentially because the cyclic PMeOx showed reduced protein binding.

#### 4.1.5 Targeted Polymer‐Lipid Considerations

In addition, the integration of stealth polymers with targeting ligands has already been successfully demonstrated in PEG‐based systems, where such combinations have enabled more selective and effective delivery across a range of biomedical applications. For example, functionalization of PEG‐LNPs with an optimally oriented anti‐Fc nanobody has been shown to increase protein expression levels by more than three orders of magnitude compared to non‐targeted formulations [96]. However, for emerging PEG alternatives, most studies to date have primarily focused on evaluating the physicochemical and delivery performance of the polymer–lipid systems themselves, often without incorporating targeting strategies. One very recent example involved the incorporation of trans‐cyclooctene functionality into PMSEA‐DSPE end groups, enabling conjugation with a tetrazine‐functionalized nanobody 9G8 for EGFR targeting. The resulting 9G8‐functionalized PMSEA mRNA‐LNPs showed enhanced cellular association and improved in vitro transfection in EGFR‐positive cells [97]. These findings highlight the potential of new polymer coatings for active targeting in mRNA‐LNP systems. Extending these approaches to include ligand‐functionalized platforms represents an important next step that could further enhance the translational potential of PEG lipid alternatives and broaden their applicability in precision medicine.

Only by broadening architectural diversity can the field fully define how polymer design controls LNP fate and function. Importantly, in the current literature on PEG‐lipid alternatives LNPs, most findings are interpreted within individual studies, where PEG‐alternative LNPs are compared with PEG‐lipid controls prepared under the same formulation conditions. Therefore, future studies should move beyond isolated comparisons of individual PEG lipid alternatives against PEG. Instead, comprehensive head‐to‐head studies should evaluate multiple stealth polymer‐lipids within the same LNP formulation platform, using identical formulation conditions, dosing routes, animal models, and biological readouts. Such matched studies will be essential to establish reliable structure–function relationships and enable clear design principles for next‐generation PEG‐alternative stealth LNPs.

We envision that the next wave of LNP innovation will rely on the rational design of “PEG‐lipid alternatives” that balance stability, immune invisibility, and biodegradability. In addition, emerging strategies such as lipid–polymer hybrid nanoparticles (LPHNPs) may offer improved pharmacokinetics and safety profiles. For instance, our recent work demonstrated that incorporating sustainable polyhydroxyalkanoate (PHA) components into conventional lipid formulations provides an alternative route to modulate particle stability and biological performance. Notably, these systems also enable the storage of mRNA therapeutics in powder form, which is highly desirable for long‐term storage and industrial translation [55].

### 4.2 Polymer Lipid Shedding

Another important but underexplored issue is whether these new polymer‐lipids remain stably associated with the LNP surface under biological conditions or even storage conditions. PEG‐lipid shedding has been shown to occur in serum and can influence LNP stability, protein adsorption, pharmacokinetics, and intracellular delivery. In conventional LNPs, PEG–lipids provide an initial steric barrier that can reduce protein adsorption and limit premature interactions with cells. However, when PEG–lipids desorb from the LNP surface, the underlying lipid components become more exposed to serum proteins. This surface remodeling can promote the adsorption of apolipoproteins, particularly ApoE, which can facilitate uptake by hepatocytes through low‐density lipoprotein receptor‐related pathways and contribute to the liver tropism commonly observed for many LNP formulations [3].

Because most PEG lipid alternatives are also introduced into LNPs through hydrophobic lipid anchors, similar surface‐dynamic processes may occur, including polymer–lipid desorption, exchange with serum lipoproteins or cell membranes, and surface rearrangement during circulation. However, such behavior should not be assumed to be identical to that of conventional PEG–lipids, as polymer chemistry, anchor structure, topology, hydration, and surface packing may alter retention and shedding kinetics. Direct and systematic measurements of surface retention or shedding remain limited for PSar‐, POx/POz‐, PMPC‐, PCB‐, PAS‐, ganglioside‐, and structurally modified PEG‐based LNP systems. As a result, it is often unclear whether improved biological performance reflects a stable stealth coating, controlled deshielding, selective protein corona formation, ApoE‐mediated uptake, or partial loss of the polymer–lipid from the nanoparticle surface.

This uncertainty is important because polymer–lipid retention and shedding may directly influence protein corona composition, organ tropism, cellular uptake, and immune recognition. Premature shedding may expose the underlying lipid surface and promote ApoE adsorption, liver accumulation, or phagocytic clearance in the liver and spleen. In contrast, controlled deshielding could be beneficial if it preserves extracellular stability. Future studies should therefore directly evaluate how polymer–lipid retention, desorption, and exchange regulate ApoE recruitment, protein corona composition, biodistribution, cell‐type‐specific uptake, intracellular trafficking, and delivery efficiency.

Specifically, current approaches to analyze PEG–lipid shedding, including labeled polymer–lipids, NMR diffusion measurements, fluorescence‐based lipid exchange assays, LC–MS quantification, and single‐particle imaging, can be useful for monitoring polymer–lipid dissociation and redistribution in serum or plasma [98]. However, many of these methods are technically demanding, indirect, or difficult to standardize across laboratories. Future studies should therefore prioritize the development of standardized assays for polymer–lipid shedding to determine whether PEG lipid alternatives function as stable stealth coatings, Ideally, these methods should allow time‐dependent quantification of polymer–lipid association with LNPs after exposure to serum, plasma proteins, lipoproteins, and cell membranes, as well as during storage.

### 4.3 Immunogenicity

It is also important to consider that PEG lipid alternatives may not be inherently non immunogenic. Indeed, our prior study showed that other POx based nanoparticles can induce anti POx antibodies [99], suggesting that antibody responses may eventually arise against most of the alternative “stealth” polymers if challenged in an appropriate model.

While PEG‐associated immune responses, including anti‐PEG antibody formation, are well documented, the immunogenic potential of the PEG lipid alternatives in LNP formulations remains largely unexplored. This raises an important open question for the field: whether PEG alternatives similarly induce antibody responses upon repeated administration of the corresponding LNPs in vivo. A deeper understanding of the long‐term immunological consequences of these materials will be critical for guiding the design of next‐generation LNPs. Nevertheless, a key current advantage of non‐PEG systems is their limited presence in everyday consumer products, such as cosmetics, toothpaste, food additives, and common medicines, unlike PEG. As a result, pre‐existing antibody levels against these alternatives in the general population are expected to be low, which may reduce the risk of immediate immune recognition and accelerated clearance upon first administration. However, this advantage depends on maintaining limited population‐level exposure. If PEG alternatives such as PSar, POx, or related polymers were to become widely used in non‐medical consumer products, similar pre‐existing antibody issues could eventually emerge. Therefore, their broader use outside biomedical applications should be carefully considered to avoid recreating the same pre‐exposure problem associated with PEG. A major unresolved challenge in the translation of PEG‐lipid alternatives in LNPs is the lack of comprehensive immunological evaluation. Although several alternative polymer–lipids have been reported to show minimal acute inflammatory responses in human or mouse blood, reduced anti‐PEG antibody binding, or limited IgG/IgM induction, these findings do not necessarily establish broad immunological safety. Specifically, anti‐PEG avoidance does not exclude the possibility of immune responses against the alternative polymer itself. Moreover, most available studies remain short‐term, single‐dose, or limited to selected immune readouts. Therefore, anti‐PEG binding, cytokine induction, complement activation, anti‐polymer antibody formation, ABC, and long‐term repeated‐dose tolerability should be considered as distinct endpoints. Table 4 and the following critical evaluation summarize how immune‐safety assessment differs across PEG‐modified and PEG‐alternative LNP systems, highlighting the need for multi‐endpoint and repeated‐dose evaluation.

**TABLE 4:** Comparative summary of immunogenicity evaluation across PEG alternatives in repeated‐dose studies.

No	PEG‐lipid alternatives class	Polymer lipid structures	Anti‐polymer antibodies measured?	ABC evaluated?	Complement / cytokines assessed?	Ref
1	PEG alteration‐lipid	PEG‐MA‐DMG and OEGMA‐DSG	Yes, mainly anti‐PEG antibody levels or anti‐PEG antibody binding	Yes, by analyzed the anti‐PEG antibody production	Not reported	[75, 77]
2	pSar‐lipids	DMG‐pSar_25_	Partly; total IgG and anti‐PEG IgG assessed, but anti‐pSar antibodies not directly measured	Not reported	Yes; mainly cytokine / chemokine induction and hepatic toxicity	[41]
3	POx / POz‐lipids	PEtOx_45_‐DMG	Yes; anti‐polymer IgM measured after repeated dosing	Yes; reduced ABC or reduced antibody‐mediated clearance reported relative to PEG‐LNPs	Not reported	[53]
4	Zwitterionic polymer‐lipids	PCB‐lipids	Yes; polymer‐specific antibody levels measured in repeated‐dose studies	Yes; ABC assessed in ALC‐0315‐based PCB‐LNPs	Yes; cytokines assessed in PBMCs, mouse repeat‐dose studies, and BALF after pulmonary delivery	[61, 70]
5	PAS‐lipid	PAS‐C16 in liposome	Restricted to anti‐PEG binding or total IgM / IgG	Not reported	Not reported	[91]

#### 4.3.1 PEG Alteration

Although PEG remains advantageous because of its more established safety profile and clinical history, immunological evaluations of PEG‐modified systems are still not always comprehensive. One strategy is to modify PEG into a brush‐like architecture. Although this approach still uses PEG, the structural alteration can change its biological behavior. After repeated administration, brush PEG‐LNPs have shown prolonged circulation compared with DMG‐PEG2000 LNPs, as indicated by higher plasma cholesteryl methyl ether levels and reduced anti‐PEG antibody levels [75, 77]. More recently, PEG isomerization has been reported as another strategy to reduce anti‐PEG antibody recognition. This effect was evaluated using enzyme‐linked immunosorbent assay (ELISA) and x‐ray reflectometry (XRR) on rPEG‐lipid monolayers designed to mimic the LNP surface, rather than on complete LNP formulations. Therefore, while PEG structural modification appears promising for reducing anti‐PEG antibody binding, further studies are needed to confirm whether these effects are retained in fully formulated LNPs under biologically relevant and repeated‐dose conditions.

#### 4.3.2 pSar

Among the limited number of pSar‐lipid LNP studies, available immunological evaluations have mainly focused on acute inflammatory readouts, including cytokine/chemokine induction and hepatic toxicity, rather than comprehensive immune safety assessment. Although one study included repeated dosing, pSar‐LNPs elicited a pro‐inflammatory pattern similar to that observed after the first dose, caused only a slight and statistically insignificant increase in total circulating IgG, and did not induce anti‐PEG IgG production, whereas PEG‐LNPs produced a stronger anti‐PEG antibody signal [41].

#### 4.3.3 POx/POz

Similarly, POx/POz‐based LNPs are among the most studied PEG‐lipid alternatives, yet their immunological evaluation remains limited. Most studies have focused on anti‐PEG antibody binding and acute cytokine responses, showing that PEtOx‐LNPs do not interact with anti‐PEG antibodies, suggesting a potential advantage for patients with pre‐existing anti‐PEG immunity. Indeed, one repeated‐dose study showed that four injections of POx/POz‐LNPs induced anti‐polymer IgM, although at significantly lower levels than those observed for PEG‐LNPs [53]. This was probably associated with reduced ABC, as fewer particles were cleared through opsonization by anti‐PEG or anti‐polymer IgM.

#### 4.3.4 Zwitterionic Polymer

A similar limitation applies to zwitterionic polymer‐lipid systems such as PCB. Current immunological evaluations have mainly focused on inflammatory responses, including cytokine secretion from human PBMCs or cytokine induction after repeat dosing in mice. For example, PCB‐LNPs based on the Moderna SM‐102 formulation showed minimal inflammatory cytokine induction, whereas PEG‐LNPs induced higher levels of pro‐inflammatory cytokines such as IL‐1β, IL‐6, TNF‐α, and IFN‐γ at higher doses compared with PCB‐LNPs [61]. In the same study, repeated administration of SM‐102‐based PCB‐LNPs generated significantly lower polymer‐specific antibody levels. This reduced antibody response was also observed in an ALC‐0315‐based formulation, where repeated administration of ALC‐0315‐PEG LNPs led to anti‐PEG IgM production and loss of efficacy, while ALC‐0315‐PCB LNPs did not show an ABC effect. These findings suggest that PCB‐LNPs may reduce antibody‐mediated ABC and improve repeated‐dose performance. In another study using PCB‐LNPs containing additional cationic lipid DOTAP for nebulized lung delivery, three repeated doses did not elevate pro‐inflammatory cytokine levels cytokines in BALF in comparison with intranasal administration of LPS [70].

#### 4.3.5 Other PEG Alternatives

For other PEG alternatives, including polypeptide‐, PAS‐, ganglioside‐, and biomimetic lipid systems, the evidence base is even more limited, often relying on only one or a small number of LNP studies. In several cases, immunological assessment has been restricted to anti‐PEG antibody binding, total IgM/IgG production, selected cytokine markers, or short‐term toxicity. For example, repeated‐dose studies of PAS‐lipid LNPs compared with PEG‐lipid LNPs showed similar blood elimination profiles, suggesting comparable pharmacokinetic behavior [91]. However, such studies remain too limited to determine whether these materials avoid anti‐polymer antibody formation or ABC over longer dosing treatment.

Overall, these examples reinforce the need for broader immunological evaluation that extends beyond a single endpoint and applies standardized, repeated‐dose assessment across PEG‐alternative LNP systems. This is particularly important because one study showed that different lipid components can trigger distinct immunogenic responses. For example, when SM‐102‐ and ALC‐0315‐based LNPs were both formulated with DMG‐pSar, some cytokines or chemokines were higher in the SM‐102‐based formulation, whereas others were lower [41]. Thus, the immunological consequences of replacing PEG‐lipids should be assessed within the same of the complete LNP formulation, including the specific ionizable lipid used. The immune response elicited by an LNP is not determined by the stealth lipid alone, but by the combined interactions among all formulation components. While PEG‐lipids appear to play an important role, definitive causation requires a deeper understanding of how PEG and other LNP components collectively drive immunogenicity [100]. Recognizing this complexity supports a more nuanced approach: rather than simply eliminating PEG‐lipids, balancing their formulation benefits with targeted mitigation of immune memory may help unlock the full potential of RNA therapeutics. This consideration is particularly important because immune stimulation may be undesirable for some therapeutic applications, but beneficial for others, such as vaccines, where activation of humoral and cellular immunity can enhance efficacy.

## 5 Conclusion

PEGylation has played a foundational role in the clinical success of LNP–based nucleic acid therapeutics. However, its immunological and pharmacokinetic liabilities increasingly limit its suitability for repeated dosing and long‐term applications. The studies reviewed here demonstrate that PEG is no longer the sole viable strategy for LNP surface engineering. A diverse array of alternative chemistries, including polysarcosine, poly(2‐oxazolines)/poly(2‐oxazines), zwitterionic polymers, structurally engineered PEG analogues, and biomimetic coatings, can reproduce key features of PEG‐mediated stealth while offering distinct advantages in immunogenicity, biodegradability, and functional performance. Importantly, these materials do not represent simple PEG mimics. Instead, they introduce new physicochemical properties and biological behaviors that have the ability to reshape protein corona formation, cellular interactions, and intracellular trafficking. The growing diversity of stealth strategies therefore marks a conceptual shift in LNP design, from reliance on a single, broadly applied polymer to a modular framework in which surface chemistry and architecture can be tailored to specific therapeutic goals. Although no single PEG‐lipid alternative yet surpasses PEG across all metrics, the collective progress reviewed here establishes a robust foundation for next‐generation LNP engineering. By integrating rational polymer design with systematic biological evaluation, the field is poised to give access to delivery systems that combine scalability with improved immune compatibility and repeat‐dose performance. Such advances will be central to the continued expansion of LNP technologies across vaccines, gene therapies, and precision nucleic acid medicines.

## Author Contributions

**Zihnil A. I. Mazrad**: Writing – original draft, Conceptualization. **Yi Ju**: Writing – review & editing, Conceptualization. **Stephen J. Kent**: Writing – review & editing, Conceptualization **Colin W. Pouton**: Writing – review & editing, Supervision, Funding acquisition, Conceptualization. **Kristian Kempe**: Writing – review & editing, Supervision, Funding acquisition, Conceptualization.

## Conflicts of Interest

The authors declare no conflicts of interest.

## Data Availability

The data that support the findings of this study are available from the corresponding author upon reasonable request.
